# YTH N6-methyladenosine RNA binding protein 2 mediated m^6^A modification of circHIPK2 promotes cellular senescence and osteoarthritis progression by inhibiting autophagy

**DOI:** 10.1186/s43556-026-00441-4

**Published:** 2026-03-27

**Authors:** Dianbo Long, Zhencan Lin, Zhiwen Li, Ming Li, Xiaoyi Zhao, Zengfa Deng, Zongrui Jiang, Wei Li, Yanlin Zhong, Aishan He, Yiyang Xu, Guping Mao, Yan Kang

**Affiliations:** 1 Department of Sports Medicine, the First Affiliated Hospital, Sun Yat-Sen University, Guangzhou, 510080 Guangdong China; 2 Department of Joint Surgery, the First Affiliated Hospital, Sun Yat-Sen University, Guangzhou, 510080 Guangdong China; 3 Guangdong Provincial Key Laboratory of Orthopedics and Traumatology, the First Affiliated Hospital, Sun Yat-Sen University, Guangzhou, 510080 Guangdong China; 4 Department of Orthopaedics, Fujian Provincial Hospital, Shengli Clinical Medical College, Fuzhou University Affiliated Provincial Hospital, Fuzhou, 350001 Fujian China

**Keywords:** CircHIPK2, N^6^‐methyladenosine, Autophagy, Chondrocyte senescence, Lipid nanoparticles

## Abstract

N6‐methyladenosine (m^6^A) modification has emerged as a critical post-transcriptional regulatory mechanism in osteoarthritis (OA). However, the contribution of m^6^A-dependent regulation of circular RNAs (circRNAs) to chondrocyte senescence and OA progression remains poorly understood. We aimed to elucidate whether m^6^A-mediated control of circRNAs regulates chondrocyte senescence and to define the underlying molecular mechanisms contributing to OA progression. Here, we identified an OA-associated circRNA, circHIPK2, and demonstrated that its abundance and function are regulated by an m^6^A reader–dependent decay mechanism. circHIPK2 expression was reduced in human OA cartilage, and its depletion exacerbated chondrocyte senescence, increased senescence-associated secretory phenotype (SASP) gene expression, and impaired autophagy both in vitro and in the destabilization of the medial meniscus (DMM) model. Mechanistically, YTH N^6^-methyladenosine RNA-binding protein 2 (YTHDF2) recognized m^6^A-modified circHIPK2 and facilitated its degradation, thereby reducing circHIPK2 stability. Functionally, circHIPK2 directly interacted with RAB22A. Loss of circHIPK2 weakened this interaction, enhanced RAB22A–PI3K association, activated the PI3K–AKT–mTOR signaling pathway, disrupted autophagic flux, and accelerated senescence-associated phenotypes. Notably, the protective effects of circHIPK2 were abolished in RAB22A-binding–deficient circHIPK2 mutants, establishing a direct link between molecular interaction and functional outcome. Furthermore, we encapsulated circHIPK2 into lipid nanoparticles (circHIPK2-LNP) for transient intra-articular delivery. Intra-articular administration of circHIPK2-LNP attenuated chondrocyte senescence and alleviated OA progression in DMM mice. Collectively, m^6^A-dependent YTHDF2-mediated degradation of circHIPK2 promotes chondrocyte senescence and OA progression by disrupting autophagy, identifying circHIPK2 as a potential therapeutic target and prognostic biomarker for cartilage aging in OA.

**Supplementary Information:**

The online version contains supplementary material available at 10.1186/s43556-026-00441-4.

## Introduction

Osteoarthritis (OA) is a chronic, painful, degenerative joint disorder characterized primarily by progressive cartilage degeneration, resulting in substantial impairment in quality of life among affected patients [1]. Disease progression is accompanied by pathological alterations in multiple joint tissues, including synovial inflammation, meniscal degeneration, and subchondral bone sclerosis, ultimately leading to persistent pain and reduced mobility, particularly in older adults [2]. The etiology of OA is multifactorial and involves complex interactions among aging, obesity, joint injury, and genetic susceptibility, with aging recognized as the most prominent risk factor [3]. Increasing evidence indicates that chondrocyte senescence represents a pivotal cellular event linking aging to cartilage extracellular matrix imbalance and structural degeneration during OA progression. Senescent chondrocytes develop a senescence-associated secretory phenotype (SASP) and secrete pro-inflammatory cytokines and catabolic factors, which accelerate cartilage breakdown and promote disease progression [4]. Accordingly, therapeutic strategies aimed at eliminating or modulating senescent cells have emerged as promising approaches for OA management [5]. However, the upstream molecular triggers and regulatory mechanisms driving chondrocyte senescence in OA remain insufficiently defined.

Autophagy is an evolutionarily conserved lysosomal degradation pathway essential for maintaining cellular homeostasis through the removal of damaged organelles and macromolecules. Its dysregulation has been implicated in multiple age-related disorders [6]. In aging cartilage, autophagic activity progressively declines in chondrocytes, leading to increased cellular stress, enhanced senescence, and accelerated cartilage degeneration [7]. Recent studies have further demonstrated that impaired autophagy promotes chondrocyte senescence and exacerbates OA progression [8]. Conversely, boosting autophagy is widely considered part of anti-aging interventions and is associated with slower OA progression [9, 10]. Despite these advances, the upstream events that lead to autophagy impairment in OA chondrocytes remain incompletely understood. Therefore, identifying key regulators that mechanistically link autophagy dysfunction to chondrocyte senescence is of particular interest.

Circular RNAs (circRNAs) are covalently closed RNA molecules characterized by high intracellular stability and emerging regulatory functions. Growing evidence supports their involvement in cartilage homeostasis and OA pathogenesis [11]. Notably, our previous studies identified several OA-associated circRNAs (circNFIX, circ0001236, and circCREBBP) that modulate chondrocyte phenotypes and cartilage degeneration, highlighting circRNAs as mechanistically informative and potentially therapeutically actionable regulators in OA [12–14]. However, for many OA-associated circRNAs, the upstream mechanisms governing their abundance and the downstream pathways through which they influence chondrocyte senescence—particularly in the context of autophagy—remain insufficiently characterized. These gaps prompted us to investigate a previously uncharacterized circRNA candidate in OA cartilage, circHIPK2. Epitranscriptomic regulation represents a plausible mechanism for the dynamic control of circRNA abundance under pathological conditions [15]. N6-methyladenosine (m^6^A) is one of the most abundant and reversible RNA modifications and plays critical roles in RNA metabolism, including structure, localization, stability, and function [16]. m^6^A modification has been implicated in the regulation of apoptosis, autophagy, and cellular senescence [17, 18], Notably, circRNAs can also harbor m^6^A marks that influence their metabolism and biological activity [11]. For example, m^6^A-modified circRNAs can be recognized by the cytoplasmic reader YTHDF2, which facilitates the formation of a circRNA–YTHDF2–HRSP12–RNase P/MRP complex, thereby promoting circRNA degradation [19]. In OA, m^6^A-modified circRNA RERE has been reported to regulate disease progression by modulating β-catenin ubiquitination and degradation [20]. Collectively, these findings suggest that m^6^A-dependent circRNA regulation may constitute an important yet underexplored mechanism contributing to the downregulation and functional loss of protective circRNAs, such as circHIPK2, in OA.

Although circRNAs are intrinsically stable within cells, their efficient intra-articular delivery remains a translational challenge. Exogenous RNAs are susceptible to degradation by synovial RNases and may be rapidly cleared by resident macrophages as well as vascular and lymphatic drainage. Therefore, the development of an effective delivery system is essential. Previous studies have demonstrated that lipid nanoparticles (LNPs) can efficiently deliver nucleic acids, including circRNA, to chondrocytes [21, 22]. Notably, intra-articular injection of FAP-targeting siRNA formulated in LNPs in murine models significantly attenuated cartilage degeneration [23]. The clinical translation of LNP technology—highlighted by the FDA approval of the first LNP-siRNA therapeutic, Onpattro®, in 2018, and the widespread application of LNP-mRNA vaccines during the COVID-19 pandemic—further underscores its potential as a robust nucleic acid delivery platform [24, 25]. Collectively, these advances provide a strong rationale for exploring LNP-based circRNA delivery strategies in OA.

In this study, we aimed to elucidate the role and regulatory mechanism of circHIPK2 in chondrocyte senescence and OA progression, with a particular focus on m^6^A-dependent epitranscriptomic regulation and autophagy regulation, and to evaluate the therapeutic potential of LNP-mediated circHIPK2 delivery. We identified a negative association between m^6^A-modified circHIPK2 levels and chondrocyte senescence and demonstrated its pivotal function in modulating OA progression in vitro and in vivo. Mechanistically, YTHDF2-mediated recognition of m^6^A modification facilitated circHIPK2 degradation in chondrocytes, thereby promoting cellular senescence through suppression of autophagy via activation of the PI3K–AKT–mTOR signaling pathway. Furthermore, intra-articular administration of circHIPK2-loaded LNPs effectively upregulated circHIPK2 expression, reduced chondrocyte senescence, and attenuated OA progression in the destabilization of the medial meniscus (DMM) mouse model. Collectively, these findings identify circHIPK2 as a promising therapeutic target for OA treatment.

## Results

### CircHIPK2 is significantly downregulated in OA tissues and its characteristics in chondrocytes

**Fig. 1:**
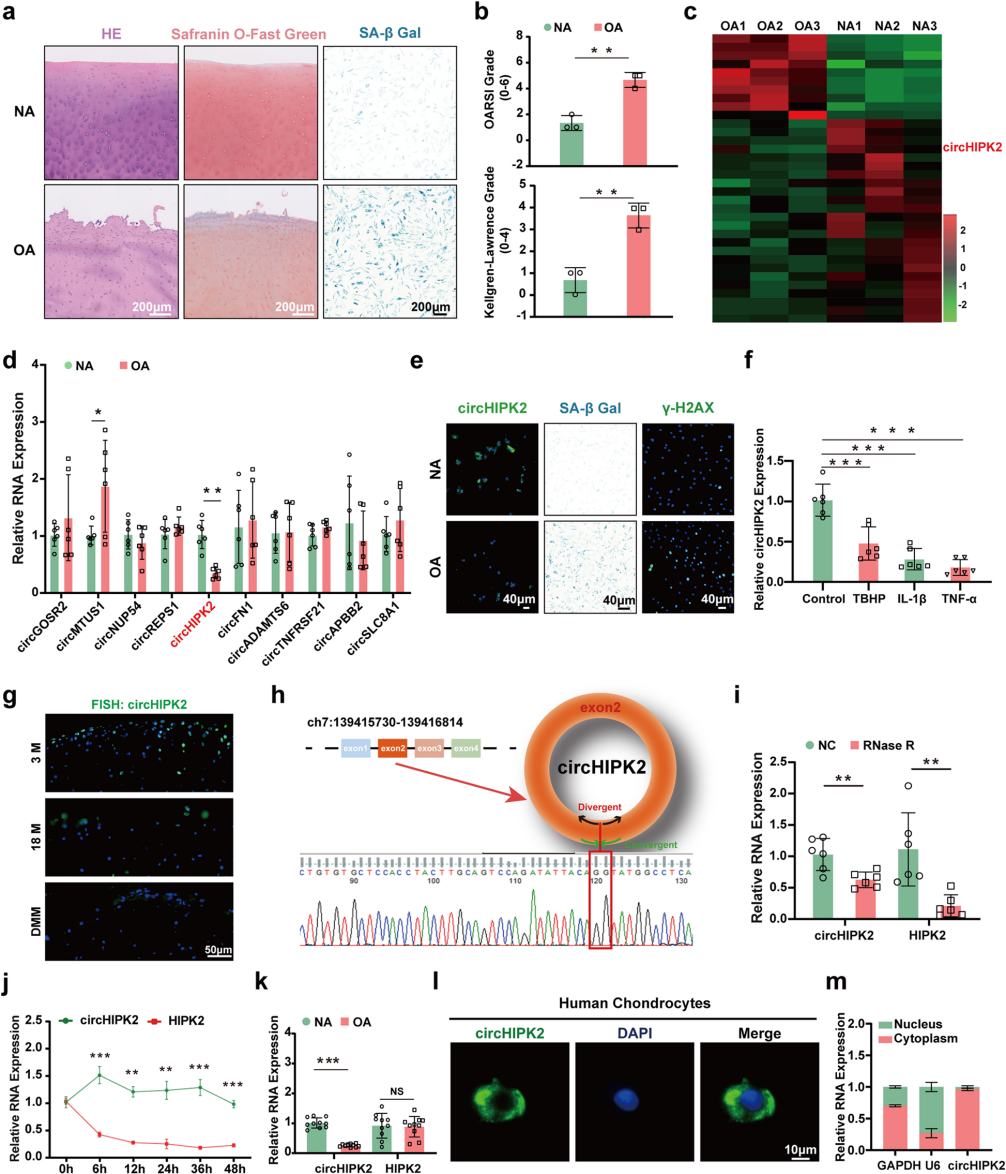
Expression pattern of circHIPK2 and its characteristics in human chondrocytes. **a**, **b** Histological analysis, preoperative Kellgren-Lawrence and OARSI grading of sequencing cartilage sample (n = 3 per group). **c** Heatmap of circRNA expression in OA and NA chondrocytes. **d** RT-qPCR analysis of circHIPK2, circGOSR2, circREPS1, circMTUS1, circNUP54, circSLC8A1, circADAMTS6, circFN1, circTNFRSF21 and circAPBB2 expression in NA and OA chondrocytes (*n* = 6 per group). **e** Representative images of circHIPK2 expression in NA and OA chondrocytes via FISH staining, γH2AX staining, SA-β gal staining. **f** CircHIPK2 expression pattern in chondrocytes treated with IL-1β, Etoposide and TNF-α, assessed via RT-qPCR (*n* = 6 per group). **g** RNA fluorescence in situ hybridization (FISH) staining of circHIPK2 in aging mice and DMM mice. **h** Schematic illustration depicting HIPK2 exons 2 circularization to form circHIPK2. The presence of circHIPK2 was validated by RT-qPCR, followed by Sanger sequencing, red box represents head-to-tail circHIPK2 splicing sites. **i** RT-qPCR analysis of circHIPK2 and HIPK2 expression in chondrocytes treated with or without RNase R (*n* = 6 per group). **j** RT-qPCR analysis of circHIPK2 and HIPK2 expression in chondrocytes treated with actinomycin D for different durations (*n* = 3 per group). **k** RT-qPCR analysis of circHIPK2 and HIPK2 expression in NA and OA chondrocytes (*n* = 10 per group). **l** Representative images of FISH staining for circHIPK2 localization. **m** RT-qPCR analysis of circHIPK2 expression in the nuclear and cytoplasmic fractions. GAPDH served as control. ^**^P < 0.01, ^***^P < 0.001

To explore differences between OA and normal (NA) cartilage, we assessed tissue degeneration using histological analysis and preoperative Kellgren–Lawrence and Osteoarthritis Research Society International (OARSI) scores. OA cartilage exhibited significantly higher degeneration than NA cartilage (Fig. 1a, b). RNA sequencing data (GSE220487) from human OA and NA cartilage tissues were analyzed, identifying 24 circRNAs with significant differential expression between the two groups (Fig. 1c; | log_2_FC (OA/NA) |> 1, FDR < 0.05). Subsequent reverse transcription–quantitative polymerase chain reaction (RT–qPCR) validation revealed that only circHIPK2 was significantly lower in OA cartilage than in NA cartilage (Fig. 1d). Additionally, immunofluorescence confirmed reduced circHIPK2 expression in OA chondrocytes (Fig. 1e, Fig. S1a). We next examined circHIPK2 expression under inflammatory and senescence-inducing conditions. Stimulation with interleukin-1β (IL-1β; 10 ng/mL), tumor necrosis factor-α (TNF-α; 50 ng/mL), or TBHP (100 μM) significantly decreased circHIPK2 expression in chondrocytes (Fig. 1f). CircHIPK2 expression was also lower in articular cartilage from 18-month-old mice and DMM mice than in that from 3-month-old controls (Fig. 1g). These findings supported the selection of circHIPK2 as a potential target for further study.

To further investigate the circular characteristics and functional role of circHIPK2, we retrieved its sequence from the circBase and circBank databases. CircHIPK2 is derived from the HIPK2 gene, located at chr7:139415730–139416814, and is formed by circular splicing of the second exon of HIPK2. Sanger sequencing confirmed the head-to-tail junction between "TACAG" and "GTATG" (Fig. 1h). Convergent and divergent primers were designed to amplify circHIPK2 from chondrocyte-derived cDNA and genomic DNA (gDNA), respectively. CircHIPK2 was amplified only by divergent primers in cDNA, confirming its circular structure (Fig. S1b). We next evaluated the stability of circHIPK2. RT–qPCR showed that circHIPK2 was resistant to RNase R digestion, whereas HIPK2 mRNA was significantly degraded (Fig. 1i, Fig. S1c). Actinomycin D treatment (0–48 h) revealed that the half-life of circHIPK2 was substantially longer than that of HIPK2 mRNA (Fig. 1j). Notably, circHIPK2 expression was significantly downregulated in OA chondrocytes, while HIPK2 mRNA levels remained unchanged (Fig. 1k). Nuclear-cytoplasmic fractionation and RNA fluorescence in situ hybridization (FISH) demonstrated that circHIPK2 is predominantly localized in the cytoplasm of chondrocytes (Fig. 1l, m). Together, these findings indicate that circHIPK2 is closely associated with chondrocyte senescence and may play a key role in OA pathogenesis.

### Knockdown of circHIPK2 accelerates cartilage degeneration and promotes chondrocyte senescence

**Fig. 2:**
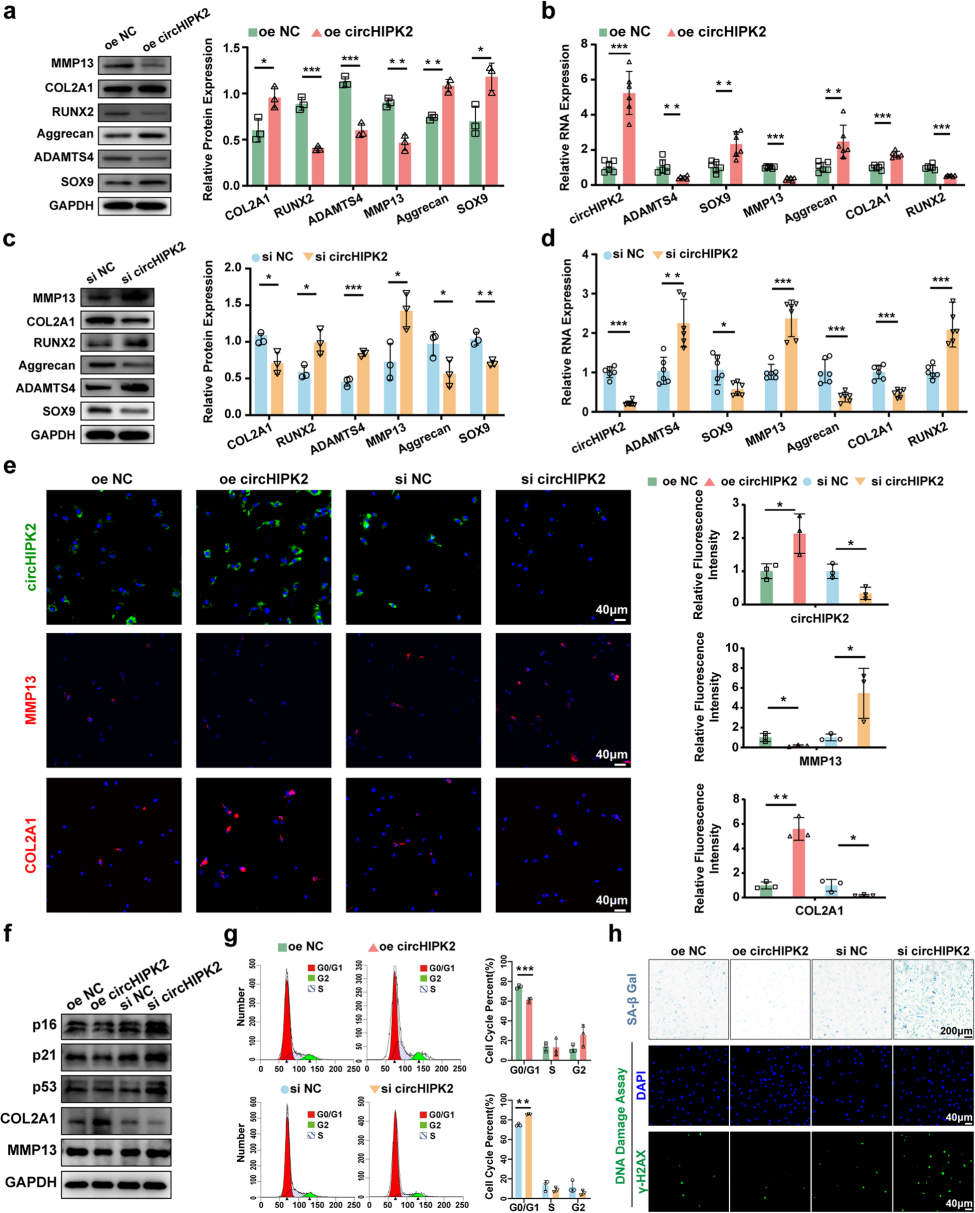
CircHIPK2 regulates cartilage degeneration and chondrocytes senescence. **a-d** Western blot and RT-qPCR analysis for the protein and mRNA expression of Aggrecan, SOX9, COL2A1, MMP13, RUNX2, and ADAMTS4 in chondrocytes transfected with oe circHIPK2 or si circHIPK2 plasmids. **e** FISH and immunofluorescence analysis of circHIPK2, MMP13, and COL2A1 levels. **f** Western blot analysis of chondrocytes degradation and senescence related proteins. **g** Cell cycle analysis of chondrocytes by flow cytometry. **h** γH2AX and SA-β gal staining were performed to evaluate chondrocyte senescence. GAPDH served as control. ^*^P < 0.05, ^**^P < 0.01, ^***^P < 0.001

To clarify the functional role of circHIPK2 in chondrocyte degeneration, primary human chondrocytes were transfected with either a circHIPK2 overexpression (oe-circHIPK2) plasmid or circHIPK2 siRNA (si-circHIPK2). Chondrocytes transfected with oe-circHIPK2 showed higher mRNA and protein levels of the anabolic markers Aggrecan, SOX9, and COL2A1, and lower levels of catabolic markers MMP13, RUNX2, and ADAMTS4 than those in normal control chondrocytes. In contrast, si-circHIPK2 transfection produced the opposite effects (Fig. 2a–d). Immunofluorescence analysis confirmed that circHIPK2 overexpression decreased MMP13 and increased COL2A1 protein expression (Fig. 2e). Notably, circHIPK2 overexpression significantly reduced the expression of senescence-associated markers p16, p21, and p53 (Fig. 2f). These molecules are well-established indicators of cellular senescence, collectively suggesting that circHIPK2 regulates chondrocyte senescence. Furthermore, SA-β-Gal and γ-H2AX staining, together with flow cytometry, confirmed that circHIPK2 overexpression suppresses chondrocyte senescence, whereas circHIPK2 knockdown promotes senescence (Fig. 2g, h). In summary, these findings demonstrate a protective role for circHIPK2 against OA progression.

### CircHIPK2 attenuates senescence-related OA pathological phenotypes

**Fig. 3:**
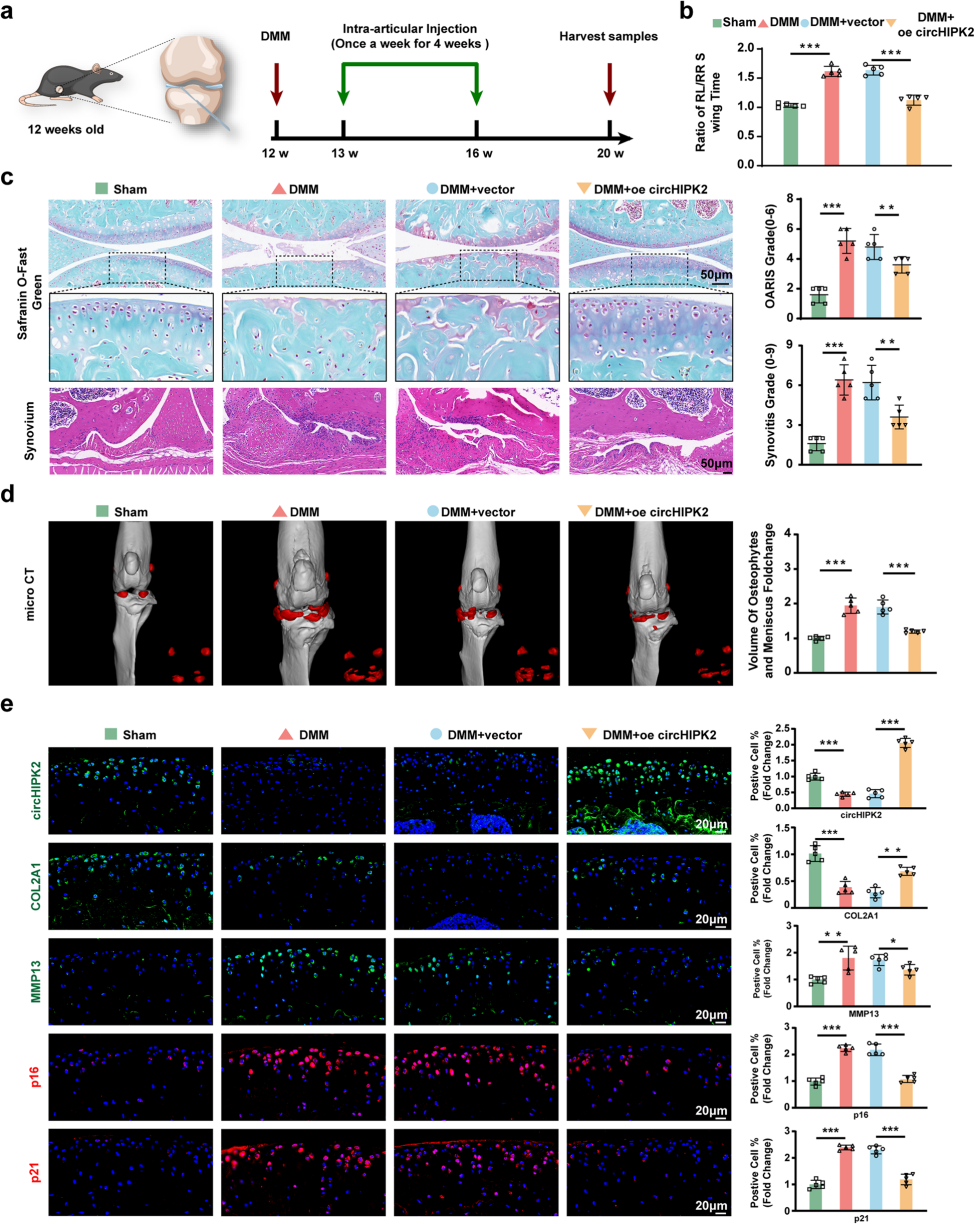
CircHIPK2 overexpression alleviates DMM-induced OA manifestations in mice. **a** Schematic depicting the DMM operation and circHIPK2 intra-articular injection in 12-week-old male C57BL/6 mice (*n* = 5 per group). One week after DMM surgery, mice received weekly intra-articular injections for four consecutive weeks. Tissues were harvested four weeks after the final dose. **b** Gait analysis showing the swing-time ratio of the left hindlimb (RL) to the right hindlimb (RR) in each group. **c** Synovial hyperplasia and cartilage degeneration evaluated by H&E and Safranin O/Fast Green staining. **d** Representative micro-CT images of calcified meniscus and osteophytes in the knee joint (red), with quantification of osteophyte volume for each group. **e** Immunofluorescence staining and FISH of circHIPK2, COL2A1, MMP13, p16 and p21 levels in the knee joints of mice in each group. ^*^P < 0.05, ^**^P < 0.01, ^***^P < 0.001

To evaluate whether intra-articular injection of circHIPK2 exerts therapeutic effects on senescence-related OA in vivo, 12-week-old male C57BL/6 mice were used to establish the DMM model and randomly assigned to four groups: sham surgery (Sham), DMM, DMM + empty adeno-associated virus (AAV) group (DMM + vector), and DMM + circHIPK2-overexpressing AAV (DMM + oe-circHIPK2). One week after DMM surgery, saline (10 μL) was injected into the Sham group, while the two DMM groups received intra-articular injections of either empty AAV (10 μL) or circHIPK2-overexpressing AAV (10 μL). Injections were administered once weekly for 4 weeks, and mice were sacrificed 8 weeks after surgery for knee specimen collection (Fig. 3a). Frozen-section analysis of knee joints revealed ZsGreen-positive chondrocytes, indicating successful transduction and expression of AAV and circHIPK2 in chondrocytes (Fig. S2a, b). Gait analysis demonstrated that the DMM and DMM + vector groups exhibited pronounced gait defects, whereas the DMM + oe-circHIPK2 group maintained normal gait patterns (Fig. 3b). Histological evaluation using hematoxylin and eosin and Safranin O/Fast Green (S–O-G) staining revealed better-preserved knee structures, lower synovitis scores, and higher proteoglycan content in the DMM + oe-circHIPK2 group than in the DMM + vector group (Fig. 3c). Micro-CT reconstruction and volumetric analysis showed fewer calcified menisci and osteophytes in the DMM + oe-circHIPK2 group (Fig. 3d). Notably, COL2A1 expression was significantly lower, while MMP13, p16, and p21 expression was significantly higher in the DMM + vector group than in the Sham group; these changes were effectively reversed by circHIPK2 overexpression (Fig. 3e, Fig. S2c, d). Collectively, these results indicate that circHIPK2 overexpression in cartilage alleviates senescence-associated manifestations in OA.

### YTHDF2 promotes m^6^A-dependent degradation of circHIPK2 in chondrocytes

**Fig. 4:**
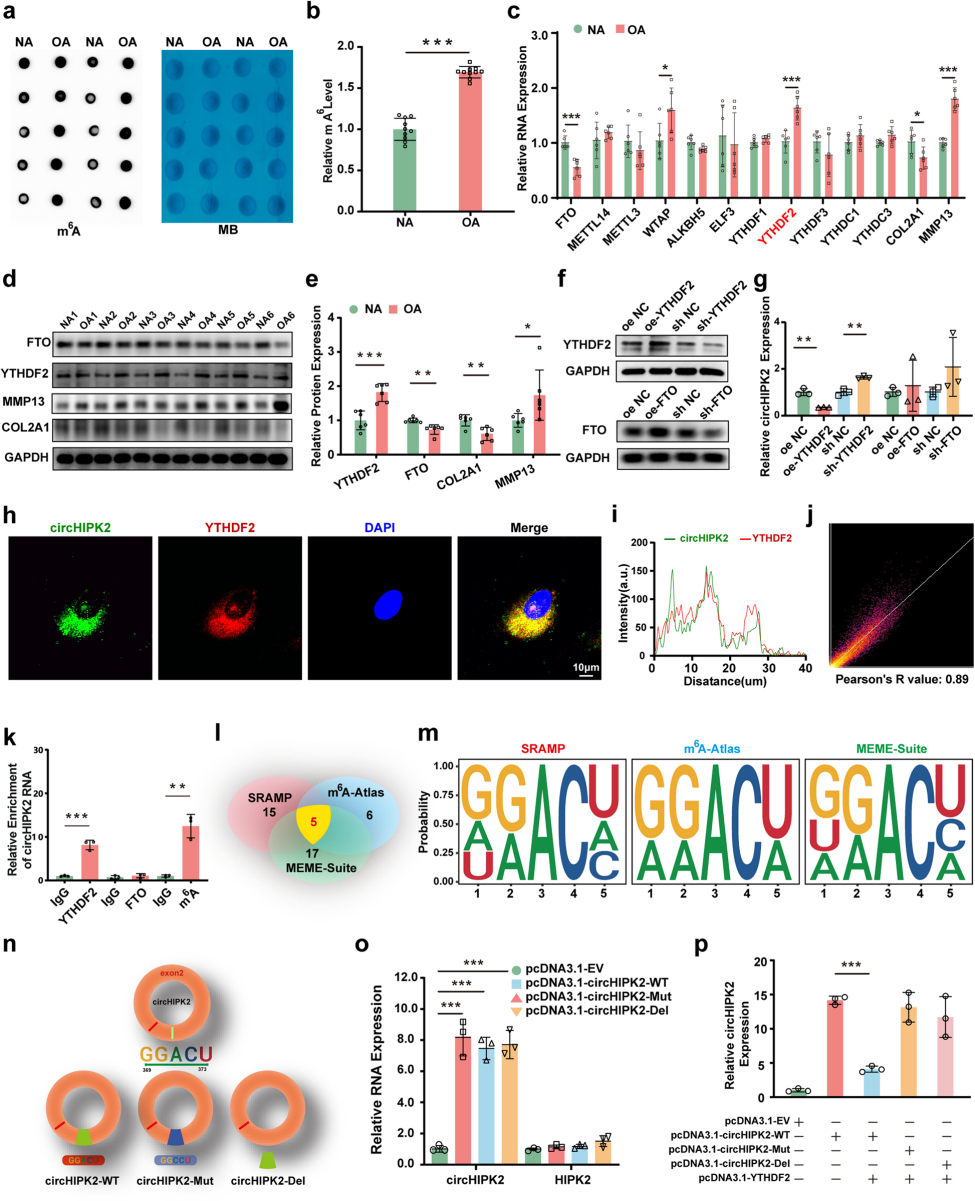
YTHDF2-mediated m^6^A modification drives the degradation of circHIPK2 in chondrocytes. **a** Dot blot analysis of total RNA m^6^A levels in OA and NA chondrocytes. **b** Quantification of dot blot analysis (*n* = 10 per group). **c** The expression of m^6^A-related genes FTO, METTL14, METTL3, WTAP, ALKBH5, eLF3, YTHDF1, YTHDF2, YTHDF3, YTHDC1, and YTHDC3 and chondrocyte degeneration-associated genes COL2A1 and MMP13 by RT-qPCR in OA and NA chondrocytes (*n* = 6). **d-e** Western blot analysis of protein levels for FTO, YTHDF2, COL2A1, and MMP13 in OA and NA chondrocytes. **f-g** Western blot analysis of YTHDF2 and FTO protein and RT-qPCR analysis of circHIPK2 in chondrocytes infected with YTHDF2 oe plasmids, YTHDF2 sh plasmids, FTO oe plasmids, FTO sh plasmids or negative control. **h-j** CircHIPK2 and YTHDF2 protein interaction in chondrocytes confirmed via RNA–protein colocalization assay. **k** m^6^A, FTO, and YTHDF2 antibody was used to detect circHIPK2 enrichment by MeRIP assay. **l-n** SRAMP, m^6^A Atlas, and MEME suite databases were used to predict methylation sites and specific information of circHIPK2. **o** RT-qPCR of circHIPK2 and HIPK2 expression in chondrocytes transfectd with pcDNA3.1-EV, pcDNA3.1-circHIPK2-WT, pcDNA3.1-circHIPK2-Mut, and pcDNA3.1-circHIPK2-Del. **p** RT-qPCR of circHIPK2 expression in chondrocytes co-transfected with pcDNA3.1-YTHDF2 and pcDNA3.1-circHIPK2-WT, pcDNA3.1-circHIPK2-Mut, and pcDNA3.1-circHIPK2-Del. GAPDH served as control. ^*^P < 0.05, ^**^P < 0.01, ^***^P < 0.001

Recent studies have highlighted the critical role of m^6^A modification in OA pathogenesis [26]. However, whether m^6^A regulates circHIPK2 function in OA remains unknown. To investigate this, we first measured global RNA m^6^A methylation levels using a dot blot assay and found that m^6^A levels were significantly higher in OA chondrocytes than in normal chondrocytes (Fig. 4a, b). Next, the expression of m^6^A-related genes—including FTO, METTL14, METTL3, WTAP, ALKBH5, eLF3, YTHDF3, YTHDF2, YTHDF1, YTHDC1, and YTHDC3—was assessed in OA and normal chondrocytes by RT–qPCR and Western blot. Among these, the m^6^A demethylase FTO and the m^6^A reader YTHDF2 showed significant differential expression between the two groups (Fig. 4c–e). To determine which m^6^A regulator specifically modulates circHIPK2, we overexpressed or knocked down FTO and YTHDF2 in chondrocytes. Only YTHDF2 overexpression significantly reduced circHIPK2 levels, whereas YTHDF2 knockdown produced the opposite effect (Fig. 4f,g). Consistently, RNA–protein colocalization analysis confirmed the physical interaction between YTHDF2 and circHIPK2 in chondrocytes (Fig. 4h-j). Furthermore, RNA immunoprecipitation (RIP) and methylated RNA immunoprecipitation (MeRIP) assays showed that circHIPK2 was significantly enriched by YTHDF2 and m^6^A antibodies, whereas no enrichment was observed in the FTO or IgG control groups (Fig. 4k). These results indicate that YTHDF2 mediates m^6^A modification of circHIPK2 and regulates its stability in chondrocytes.

To further identify the m^6^A methylation sites on circHIPK2, we used the SRAMP, m^6^A Atlas, and MEME Suite databases to predict potential modification sites and analyzed the overlapping regions using HOMER motif analysis. This analysis revealed that the motif GGACU at bases 369–373 of circHIPK2 was the region with the highest probability of m^6^A modification (Fig. 4l, m). Based on this sequence, three circHIPK2 plasmids were constructed: wild-type (pcDNA3.1-circHIPK2-WT), GGACU mutated to GGCCU (pcDNA3.1-circHIPK2-Mut), and GGACU-deleted plasmid (pcDNA3.1-circHIPK2-Del) (Fig. 4n). All plasmids were successfully transfected into chondrocytes. RT–qPCR analysis showed that circHIPK2 expression was higher in all three plasmid-transfected groups than in controls, whereas HIPK2 mRNA levels remained unchanged, indicating that mutation or deletion of the motif did not disrupt the circular structure of circHIPK2 (Fig. 4o). Subsequently, chondrocytes were co-transfected with the YTHDF2 overexpression plasmid (pcDNA3.1-YTHDF2) and each of the three circHIPK2 plasmids. In the pcDNA3.1-YTHDF2 + pcDNA3.1-circHIPK2-WT group, circHIPK2 expression was significantly lower than that in the pcDNA3.1-circHIPK2-WT group. In contrast, circHIPK2 levels in the pcDNA3.1-YTHDF2 + pcDNA3.1-circHIPK2-Mut and pcDNA3.1-YTHDF2 + pcDNA3.1-circHIPK2-Del groups were not significantly affected (Fig. 4p).

Based on our in vitro findings demonstrating that YTHDF2 mediates m^6^A-dependent degradation of circHIPK2, thereby contributing to OA-related chondrocyte phenotypes, we next investigated whether targeting YTHDF2 in vivo would validate the pathophysiological relevance of this regulatory axis. To this end, 12-week-old male C57BL/6 mice underwent DMM surgery, followed 1 week later by intra-articular injection of saline (Sham), control vector, or shYTHDF2 AAV. Injections were administered weekly for 4 consecutive weeks. Mice were sacrificed 8 weeks after DMM surgery, and knee specimens were collected for analysis (Fig. S3a). Gait analysis and S–O-G staining revealed that mice treated with DMM + shYTHDF2 AAV exhibited improved gait patterns and better preservation of cartilage structure than those in the DMM + vector group (Fig. S3b, c). Consistently, micro-CT reconstruction revealed that shYTHDF2 AAV treatment effectively reduced calcified menisci formation and osteophyte development in DMM mice (Fig. S3d). Notably, intra-articular administration of shYTHDF2 AAV increased circHIPK2 expression and alleviated cartilage pathological changes in DMM mice, as evidenced by elevated COL2A1 expression and reduced MMP13 and p21 expression (Fig. S3e). Collectively, these findings indicate that YTHDF2 promotes OA progression by targeting the m^6^A-modified site of circHIPK2, leading to its downregulation in vivo. Given that YTHDF2-driven m^6^A-dependent degradation reduces circHIPK2 abundance, we hypothesized that this mechanism may impair circHIPK2’s downstream protective functions by limiting its interaction with key effector proteins. Therefore, we next investigated whether circHIPK2 depletion attenuates its interaction with RAB22A.

### CircHIPK2 exerts its biological functions by directly binding to RAB22A protein in chondrocytes

**Fig. 5:**
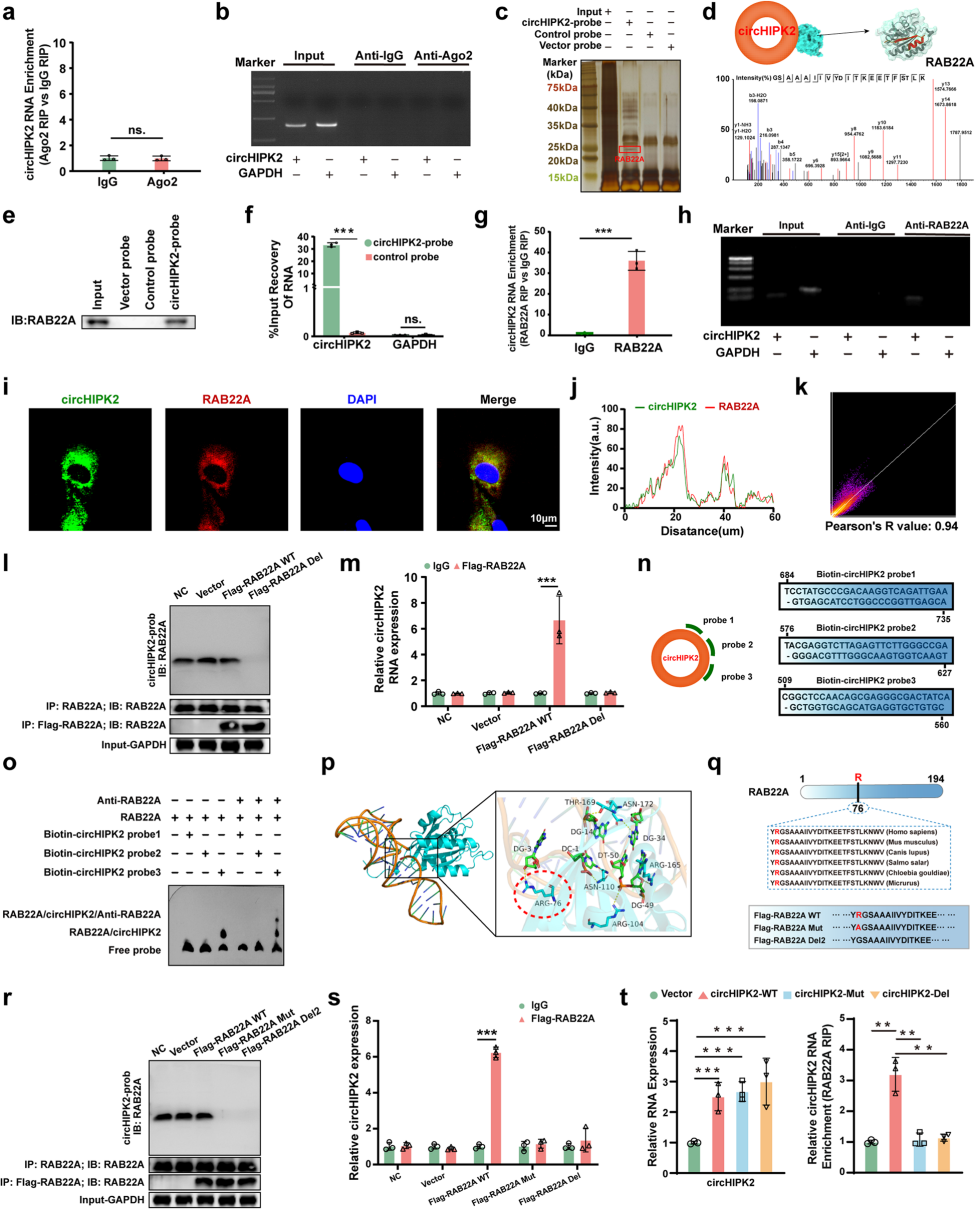
CircHIPK2 directly binds to RAB22A in chondrocytes. **a** Ago2 antibody was used to assess circHIPK2 and Ago2 protein interaction by RIP assay. **b** RT-qPCR products showing no-interaction between circHIPK2 and Ago2 protein. **c** Vector probe, control probe, and circHIPK2 probe were employed for pulldown assay to identify circHIPK2-binding proteins. **d** Mass spectrometry results revealed that circHIPK2 bound to RAB22A protein. **e–f** Immunoblot analysis to determine RAB22A protein expression in the blank probe group, control probe group, and circHIPK2 probe group. RT-qPCR was utilized to evaluate circHIPK2 enrichment by the circHIPK2 probe. **g-h** RIP assay analysis of circHIPK2 and RAB22A protein interaction. **i-k** CircHIPK2 and RAB22A protein interaction in chondrocytes confirmed via an RNA–protein colocalization assay. **l-m** Following transfection of pcDNA3.1-Vector, Flag-RAB22A-WT, Flag-RAB22A-Del (76–97) into chondrocytes, use pulldown assay and RIP assay to identify circHIPK2 interacts with RAB22A proteins. **n–o** Direct interaction between circHIPK2 and RAB22A identified by Electrophoretic mobility shift assay (EMSA). **p** Molecular docking analysis between circHIPK2 and RAB22A protein. **q-s** Following transfection of pcDNA3.1-Vector, Flag-RAB22A-WT, Flag-RAB22A-Mut (R76A), Flag-RAB22A-Del2 into chondrocytes, use pulldown assay and RIP assay to identify binding domain of circHIPK2 and RAB22A proteins. **t** Following transfection of Vector, circHIPK2-WT, circHIPK2-Mut (G511A), and circHIPK2-Del (lacking G511) into chondrocytes, RIP assay to identify circHIPK2 interacts with RAB22A proteins. GAPDH served as control. ^**^P < 0.01, ^***^P < 0.001, ns: no significance

CircRNAs exert their biological functions primarily through competing endogenous RNA (ceRNA) activity, protein binding, and peptide translation [27]. To determine the functional mechanism of circHIPK2, we first performed RIP using an Ago2 antibody to assess its potential involvement in a ceRNA network. No significant enrichment of circHIPK2 was observed in the Ago2 group compared with the IgG control (Fig. 5a, b), suggesting that circHIPK2 is unlikely to function through a canonical miRNA-mediated mechanism. Subsequent analysis using the circBank database and circPrimer2.0 software indicated that circHIPK2 lacks strong translation potential (Fig. S4a). Therefore, we hypothesized that circHIPK2 may function through protein binding. An RNA pull-down assay using a biotin-labeled circHIPK2 probe revealed that circHIPK2 interacts with multiple proteins, with the strongest binding affinity observed for RAB22A (Fig. 5c, d). Immunoblotting analysis confirmed the interaction between RAB22A and circHIPK2 (Fig. 5e, f). Consistently, both the circHIPK2 probe and the RAB22A antibody significantly enriched circHIPK2 in RIP assays (Fig. 5g, h). RNA–protein colocalization analysis in chondrocytes further verified the interaction between circHIPK2 and RAB22A (Fig. 5i-k). Guided by CatRAPID predictions (Fig. S4b, c), we generated Flag-RAB22A-WT and a deletion mutant, Flag-RAB22A-Del (76–97). Pull‐down and RIP assays demonstrated that deletion of residues 76–97 abolished the circHIPK2–RAB22A interaction (Fig. 5l, m). To further delineate the binding interface, electrophoretic mobility shift assays (EMSA) were performed using three circHIPK2 probes (probe 1: 684–735; probe 2: 576–627; probe 3: 509–560). Only the 509–560 region of circHIPK2 directly interacted with the 76–97 region of RAB22A (Fig. 5n, o). Molecular docking analysis predicted a high-affinity interaction between nucleotide G511 of circHIPK2 and residue R76 of RAB22A (Fig. 5p). Accordingly, we constructed Flag-tagged RAB22A variants, including wild-type (Flag-RAB22A-WT), a point mutant (Flag-RAB22A-Mut, R76A), and a deletion mutant lacking residue 76 (Flag-RAB22A-Del2). Both pull-down and RIP assays confirmed that mutation or deletion of residue 76 significantly impaired the circHIPK2–RAB22A interaction (Fig. 5q–s).

To further validate the binding specificity from the circHIPK2 side, we generated circHIPK2 constructs including circHIPK2-WT, circHIPK2-Mut (G511A), and circHIPK2-Del (ΔG511). All constructs effectively increased circHIPK2 expression, indicating that mutation or deletion at the G511 locus did not disrupt circHIPK2 circularization or stability. Notably, RIP assays demonstrated that either mutation or deletion of G511 significantly reduced RAB22A binding (Fig. 5t).

Collectively, these findings identify G511 of circHIPK2 and R76 of RAB22A as the critical residues mediating their interaction and confirm the specificity and functional relevance of the circHIPK2–RAB22A interface.

### CircHIPK2-RAB22A axis regulates autophagy by PI3K-AKT-mTOR signaling pathway to alleviate chondrocyte senescence

**Fig. 6:**
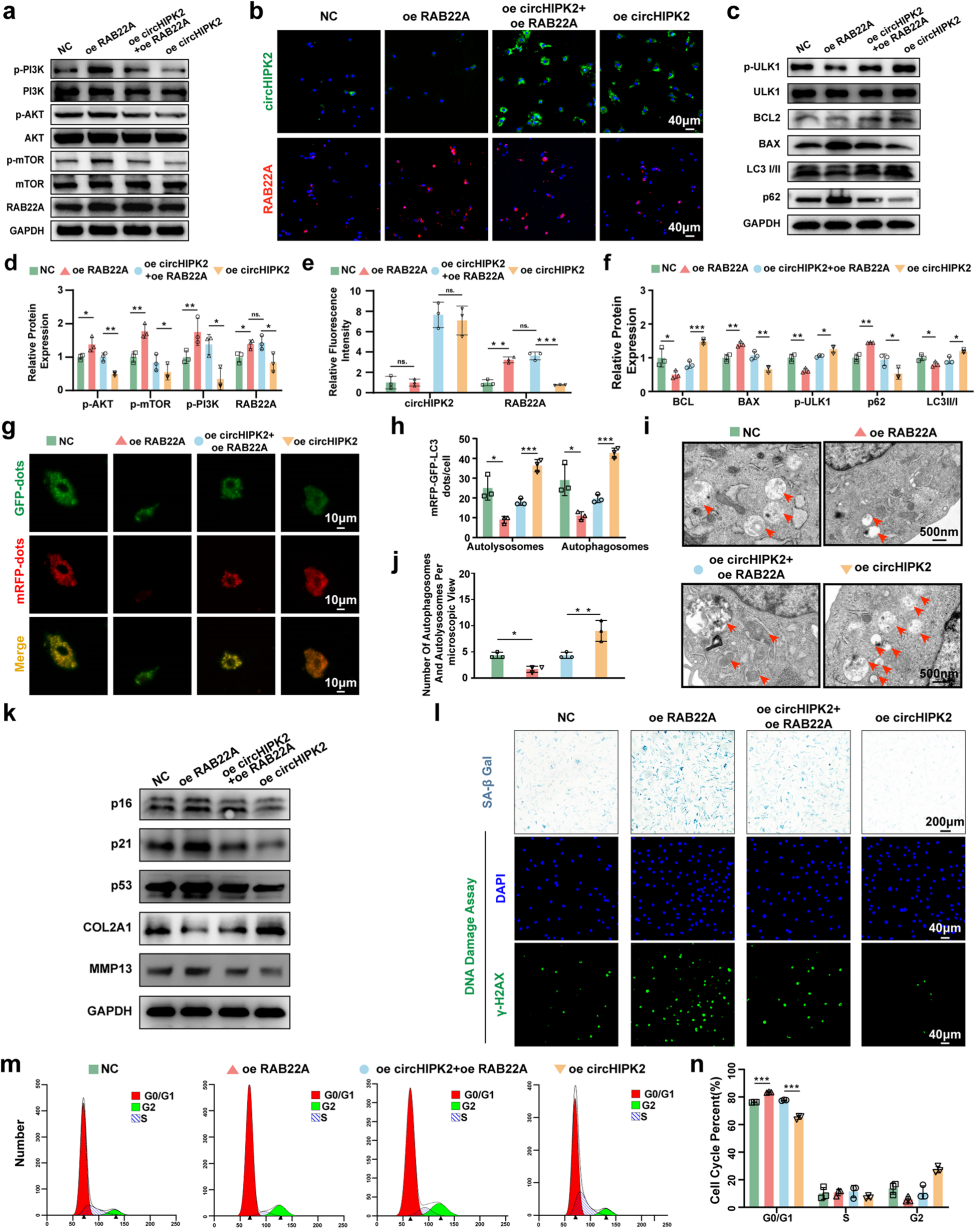
RAB22A overexpression inhibited autophagy and promoted chondrocytes senescence through PI3K-AKT-mTOR pathway. Chondrocytes were transfected with circHIPK2 oe plasmids, RAB22A oe plasmids or negative control. **a** Western blot analysis of p-PI3K, p-AKT, p-mTOR, PI3K, AKT, and mTOR proteins. **b** FISH and immunofluorescence analysis of circHIPK2 and RAB22A. **c** Western blot analysis of autophagy-associated proteins p-ULK1, ULK1, P62, LC3 I/II, BCL2, and BAX. **d-f** Quantification of western blot, FISH and immunofluorescence analysis. **g-h** mRFP-GFP-LC3 double-labeled autophagy adenovirus was employed to assess the changes of autophagic flux in chondrocytes. **i-j** Representative TEM images of autophagosomes and autolysosomes in chondrocytes. Green arrows: autophagosomes, red arrows: autolysosomes. **k** Western blot analysis of chondrocytes extracellular matrix-associated proteins and senescence-associated proteins. **l** γH2AX and SA-β gal staining of chondrocytes. **m–n** Cell cycle analysis of chondrocytes by flow cytometry. GAPDH served as control. ^*^P < 0.05, ^**^P < 0.01, ^***^P < 0.001, ns: no significance

RAB22A has been reported to promote PI3K phosphorylation and activate the PI3K–AKT–mTOR signaling pathway, thereby inhibiting autophagy [28]. Based on this evidence, we investigated the specific role of RAB22A in regulating chondrocyte autophagy during OA progression. RAB22A overexpression significantly increased the levels of p-PI3K, p-AKT, p-mTOR, p-ULK1, LC3 II/I, and p62, whereas these effects were effectively reversed by circHIPK2 overexpression (Fig. 6a–f). These findings prompted us to hypothesize that circHIPK2 may modulate the interaction between RAB22A and PI3K. To test this, we performed proximity ligation assays in chondrocytes. The results showed that the RAB22A–PI3K interaction was significantly higher in OA chondrocytes than in normal controls (Fig. S5a). CircHIPK2 overexpression reduced this interaction, whereas circHIPK2 knockdown further enhanced it (Fig. S5b). Consistently, transfection with wild-type circHIPK2 attenuated RAB22A–PI3K binding, whereas circHIPK2-Mut and circHIPK2-Del constructs had no significant effect (Fig. S5c). Together with our binding-site mapping data (Fig. 5l–t), these results indicate that circHIPK2 must directly bind RAB22A to antagonize the RAB22A–PI3K interaction and exert its functional effects.

To further assess autophagic activity, mRFP–GFP–LC3 adenoviral double labeling and transmission electron microscopy (TEM) were performed. RAB22A overexpression reduced the numbers of autolysosomes and autophagosomes, whereas circHIPK2 overexpression restored these structures (Fig. 6g–j). These findings confirm that the circHIPK2–RAB22A axis regulates autophagy in chondrocytes. Notably, RAB22A overexpression significantly increased the expression of p16, p21, p53, and MMP13 in human chondrocytes (Fig. 6k), accompanied by an increased proportion of senescent chondrocytes as demonstrated by SA-β-Gal staining, γ-H2AX staining (Fig. 6l), and flow cytometry (Fig. 6m, n). CircHIPK2 overexpression effectively rescued the RAB22A-induced senescence phenotype (Fig. 6k–n). To further clarify the stage at which autophagy was inhibited, cells were treated with bafilomycin A1 (BafA1), which blocks lysosomal degradation and causes LC3-II and p62 accumulation. Compared with BafA1 treatment alone, RAB22A overexpression did not further increase LC3-II or p62 levels in the presence of BafA1. These findings indicate that RAB22A inhibits autophagy at an upstream stage—specifically at the level of autophagosome formation—rather than merely blocking lysosomal degradation. Co-expression of circHIPK2 with RAB22A restored LC3-II accumulation and reduced p62 levels, consistent with recovery of autophagic flux (Fig. S5d). Conversely, in chondrocytes co-transfected with shRAB22A and si-circHIPK2, the senescence-attenuating effect of RAB22A knockdown was reversed by circHIPK2 silencing (Fig. S6a–n), further supporting the functional interdependence of this regulatory axis.

Taken together, these findings demonstrate that circHIPK2 suppresses chondrocyte senescence by directly binding RAB22A, thereby disrupting the RAB22A–PI3K interaction, inhibiting PI3K–AKT–mTOR signaling, enhancing autophagy, and ultimately attenuating OA progression.

### Synthesis and characterization of circHIPK2-LNP

Although AAV enables stable and long-term circHIPK2 expression for mechanistic validation, its clinical application is limited by challenges in precise dose control and minimizing off-target effects. Furthermore, despite the intrinsic stability of circHIPK2 and its resistance to intracellular RNase-mediated degradation, effective in vivo delivery remains challenging due to exposure to synovial nucleases, rapid clearance, and limited cellular uptake. LNPs have emerged as a leading nanocarrier platform for therapeutic RNA delivery because of their favorable encapsulation efficiency, protection against nuclease degradation, and capacity for cellular internalization [21–23]. Therefore, we developed an LNP-based delivery system to better simulate a clinically translatable strategy with transient and controlled circHIPK2 administration. First, circHIPK2 was successfully synthesized and validated in vitro. RNase R digestion assays demonstrated that the in vitro-transcribed circHIPK2 was resistant to exonuclease degradation. Sanger sequencing verified the back-splice junction, confirming accurate circularization at the designated site. Additionally, Agilent 2100 Bioanalyzer analysis revealed high RNA integrity without detectable degradation or nonspecific bands (Fig. S7a). Subsequently, circHIPK2 was encapsulated into LNPs (Fig. S7b). TEM revealed uniformly distributed, spherical circHIPK2-LNP particles (Fig. S7c). Dynamic light scattering analysis showed an average particle size of 70.06 nm with a polydispersity index of 0.05, indicating a highly uniform size distribution. The mean zeta potential was − 3.94 mV (Fig. S7d). Using a dose range of 50–200 ng circHIPK2-LNP, sustained fluorescence was detected in chondrocytes for up to 72 h, with the 200 ng dose exhibiting the longest detectable signal (Fig. S7e). Cell viability and cytotoxicity assays demonstrated minimal toxicity of circHIPK2-LNP in vitro (Fig. S7f, g). To further evaluate in vivo biosafety, mice received intra-articular injections of either phosphate-buffered saline (PBS) or circHIPK2-LNP. At 4 weeks post-injection, histopathological examination revealed normal morphology of the heart, lungs, liver, spleen, and kidneys in both groups (Fig. S7h). At 7 days post-injection, routine hematological and biochemical parameters remained within normal ranges, with no significant differences between groups (Fig. S7i). Collectively, these findings demonstrate that injectable circHIPK2-LNP represents a safe and effective strategy for delivering circHIPK2 to cartilage in vivo.

### CircHIPK2-LNP treatment alleviates OA progression in DMM mice

**Fig. 7:**
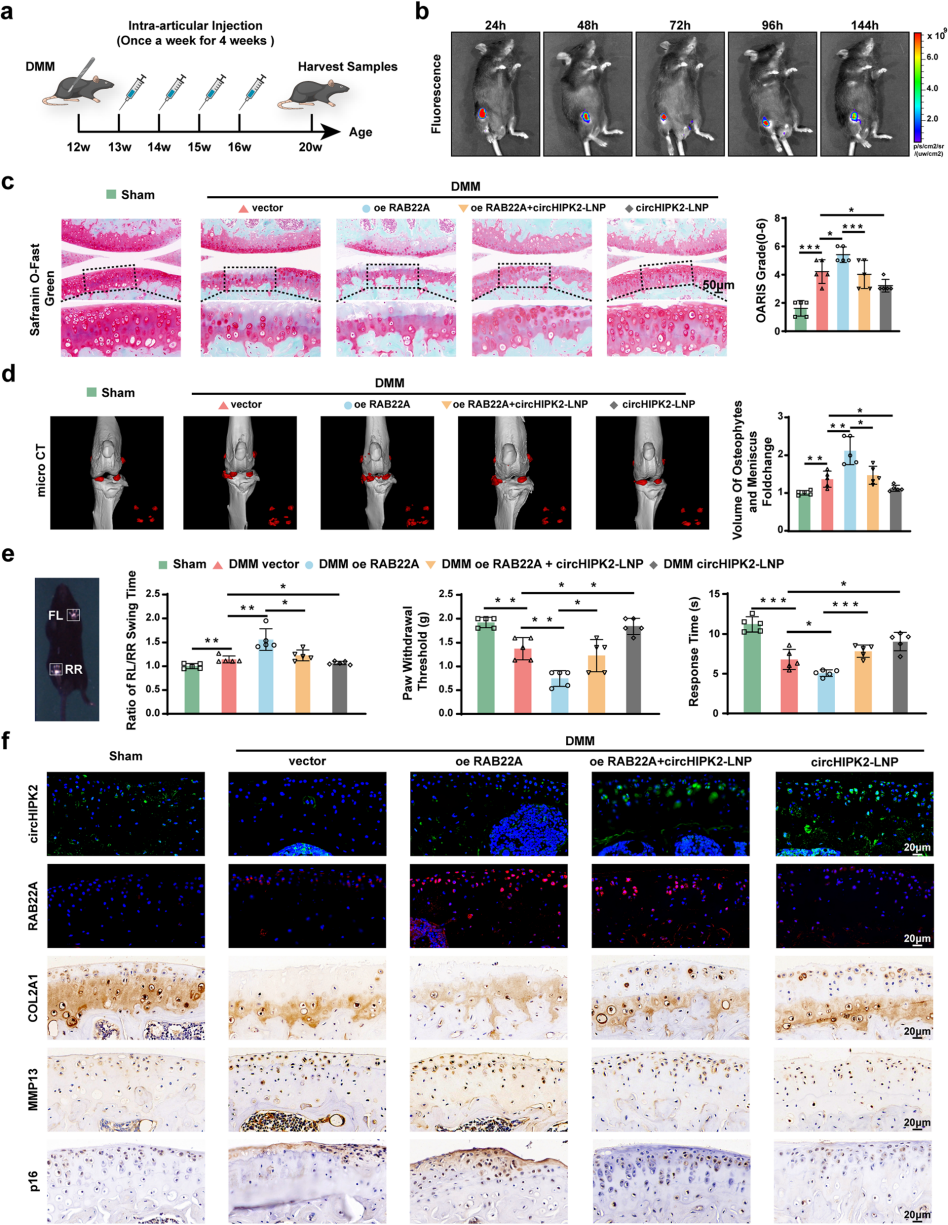
Therapeutic effect of circHIPK2-LNP in DMM mice. **a** Schematic depicting the DMM operation and circHIPK2-LNP intra-articular injection in 12-week-old male C57BL/6 mice (*n* = 5 per group). One week after DMM surgery, mice received intra-articular injections weekly for four consecutive weeks. Samples were harvested four weeks after the final injection. **b** Bioluminescence imaging at different time points after intra-articular injection of cy5-circHIPK2-LNP in mouse knee joints. **c** The extent of cartilage degeneration assessed by Safranin-O fast green staining and quantification of cartilage degeneration by OARSI grade. **d** Micro-CT images of knee joint calcified meniscus and osteophytes (Red) and quantification of the volume in each group. **e** Ratio of rear left (RL) to rear right (RR) limb swing time of mice in each group according to gait analysis. Pain hypersensitivity levels were assessed by Von Frey assays and Hotplate pain assay. **f** Immunohistochemistry staining, immunofluorescence staining and FISH of circHIPK2, RAB22A, COL2A1, MMP13, and p16 in the knee joints of mice in each group. ^*^P < 0.05, ^**^P < 0.01, ^***^P < 0.001

To further evaluate the therapeutic efficacy of circHIPK2-LNP based on the circHIPK2–RAB22A axis identified in our previous experiments, we established the DMM model in 12-week-old male C57BL/6 mice. The mice were randomly assigned to five groups: Sham, DMM + empty AAV (DMM + vector), DMM + RAB22A overexpression AAV group (DMM oe-RAB22A), circHIPK2-LNP overexpression group (circHIPK2-LNP), and DMM + RAB22A overexpression AAV + circHIPK2-LNP overexpression group (DMM oe-RAB22A + circHIPK2-LNP). One week after DMM surgery, equal doses of the corresponding AAV constructs or nanoparticles were intra-articularly injected into the knee joint of mice in each group. Injections were administered once weekly for 4 consecutive weeks. All mice were sacrificed at 20 weeks of age for specimen collection (Fig. 7a). In vivo imaging demonstrated that circHIPK2-LNP sustained localized circHIPK2 expression in the knee joint for at least 144 h (Fig. 7b). RAB22A overexpression induced synovial hypertrophy and hyperplasia, increased the thickness of the synovial lining cell layer, and significantly exacerbated cartilage destruction. These pathological changes were reversed by circHIPK2 overexpression (Fig. 7c, Fig. S8a). Micro-CT reconstruction and quantitative volumetric analysis revealed fewer calcified menisci and osteophytes in the DMM oe RAB22A + circHIPK2-LNP group (Fig. 7d). Gait analysis showed that the rear left/rear right swing time ratio was significantly higher in the DMM + oe-RAB22A group than in the DMM + vector group, whereas this alteration was ameliorated in the circHIPK2-LNP-treated group. Notably, circHIPK2 overexpression also alleviated DMM-induced OA-associated pain (Fig. 7e). Immunohistochemistry and immunofluorescence analyses of joint sections demonstrated that circHIPK2 overexpression increased COL2A1 expression while reducing MMP13 and p16 levels (Fig. 7f, Fig. S8b). Collectively, these findings indicate that circHIPK2-LNP exhibits anti-senescence effects by inhibiting RAB22A and restoring cartilage homeostasis in vivo, thereby supporting the therapeutic potential of circHIPK2 for the treatment of senescence-related OA.

## Discussion

OA is the most common age-related joint disorder and currently lacks disease-modifying therapies. As the unique cell type in articular cartilage, chondrocyte senescence represents a pivotal cellular event contributing to OA pathogenesis. In this study, we demonstrate that circHIPK2 exerts chondroprotective effects during OA progression, and its overexpression inhibits the senescent phenotype. Mechanistically, YTHDF2-mediated m^6^A modification of circHIPK2 promotes cellular senescence and OA progression by reducing its interactions with RAB22A, thereby inhibiting chondrocyte autophagy through activation of the PI3K–AKT–mTOR pathway.

m^6^A modification is the most prevalent post-transcriptional modification in mRNA and non-coding RNA and has recently been identified in circRNAs as well [29, 30]. Emerging evidence indicates that m^6^A modifications are recognized by YTH domain family proteins, including YTHDF1, YTHDF2, and YTHDF3, and m^6^A-modified circRNAs can be degraded in a YTHDF2-dependent manner [31–33]. In our study, m^6^A modification was enhanced during OA progression, accompanied by increased YTHDF2 expression, whereas YTHDF1 and YTHDF3 levels remained unchanged. Bioinformatic prediction combined with experimental validation demonstrated that m^6^A modification of circHIPK2 was specifically regulated by YTHDF2, but not by YTHDF1 or YTHDF3. Notably, YTHDF2-mediated m^6^A modification promoted circHIPK2 degradation. Consistent with our findings, previous studies have reported that YTHDF2 facilitates circHIPK2 degradation in the mouse cortex and BV2 cells [34]. Our results further support a YTHDF2-dependent degradation mechanism for m^6^A-modified RNAs and highlight m^6^A as a potential selective regulatory signal governing circRNA metabolism. We also observed that FTO was significantly downregulated in OA. However, FTO knockdown or overexpression did not alter circHIPK2 expression, suggesting that FTO may not directly regulate circHIPK2 in this context. The precise role of FTO in OA pathogenesis warrants further investigation.

CircRNAs primarily exert their biological functions through four well-established mechanisms: (1) acting as competitive endogenous RNAs (ceRNAs) by binding miRNAs [13], as reported for circHIPK2 in astrocytes [35]; (2) regulating parental gene expression through alternative splicing [19]; (3) encoding novel proteins [36]; and (4) interacting with RNA-binding proteins to form RNA–protein complexes, a mechanism more recently characterized [37]. Our previous studies demonstrated that several circRNAs (circNFIX, circ0001236, and circCREBBP) regulate chondrocyte differentiation via classical miRNA-sponging mechanisms, thereby promoting OA progression [12–14]. In contrast, this study reveals for the first time that circHIPK2 directly promotes chondrocyte senescence through an RNA-binding protein–dependent mechanism. We found that circHIPK2 degradation weakens its direct interaction with RAB22A, thereby activating the PI3K–AKT–mTOR pathway, inhibiting chondrocyte autophagy, and accelerating cellular senescence during OA progression. Defective autophagy has been reported to drive senescence in various pathological conditions, including pulmonary fibrosis, cancer, aging-related disorders, and cardiovascular diseases [38]. Conversely, enhanced autophagy helps maintain bone metabolic homeostasis and attenuates cartilage degeneration in OA [39]. Although the PI3K–AKT–mTOR pathway is well recognized as a key regulator of chondrocyte autophagy and OA progression, its upstream modulators have largely been identified as proteins or pharmacological agents [40, 41]. Unlike the extensively studied roles of miRNAs and long non-coding RNAs in autophagy regulation [42], our findings identify circHIPK2 as a novel upstream RNA regulator of the PI3K–AKT–mTOR pathway, highlighting its critical role in modulating autophagy during OA development.

To date, the FDA has approved four LNP-based RNA therapeutics. Compared with other RNA delivery platforms, such as viral vectors or extracellular vesicle-based carriers, LNPs offer substantial advantages in terms of cost-effectiveness, safety, flexibility, and ease of chemical modification, making them highly suitable for clinical translation [43, 44]. Furthermore, circRNAs exhibit greater structural stability and longer intracellular persistence than siRNA or linear mRNAs, which may enable more sustained therapeutic effects within the joint even when delivered via an LNP system [45, 46]. Together, the combination of circHIPK2 and LNP delivery represents a promising strategy for OA therapy by optimizing safety, durability, and translational feasibility. In this study, we developed chondrocyte-targeting circHIPK2-LNP and validated their efficacy and safety in vivo. Notably, intra-articular administration of circHIPK2-LNP significantly alleviated chondrocyte senescence and slowed OA progression in the DMM model.

This study has some limitations. First, although we identified and characterized RAB22A as a key interacting partner of circHIPK2, the possibility of additional binding partners and other downstream signaling pathways in chondrocytes remains to be explored. Second, the potential for off-target effects of the LNP delivery system or circHIPK2 itself requires further investigation. The lipid compositions of LNPs may elicit unintended immune responses, potentially compromising therapeutic efficacy. Similarly, exogenous circHIPK2 could non-specifically interact with other miRNAs or cellular proteins, which may inadvertently disrupt critical chondrocyte functions. Third, while the DMM model provides valuable mechanistic insights, it does not fully recapitulate human OA. Future studies across multiple species and models will be essential to validate and extend these findings.

In conclusion, this study provides the first comprehensive evidence that m^6^A modification of circHIPK2 plays a critical role in OA. CircHIPK2 promotes chondrocyte autophagy by forming a circHIPK2–RAB22A RNA–protein complex that regulates the PI3K–AKT–mTOR pathway. Furthermore, the circHIPK2-LNP delivery system represents a promising platform for disease-modifying OA drugs, offering an innovative therapeutic approach for OA treatment.

## Materials and methods

### Tissue collection and chondrocytes isolation and culture

Human cartilage tissues were obtained from patients undergoing total knee arthroplasty. According to the International Cartilage Repair Society (ICRS) grading system, knee cartilage was categorized into intact areas (ICRS = 0) and worn areas (ICRS = 3–4), which were collected separately. In this study, chondrocytes from the intact areas without wear were defined as normal articular cartilage (NA), whereas OA cartilage were from worn areas (ICRS = 3–4). We also collected patient characteristics, including radiography, age, sex, affected side, OARSI Grade and Kellgren-Lawrence grade. Baseline of characteristics patients with osteoarthritis presented at Table S3. Cartilage specimens were dissected from the subchondral bone and cut into small pieces. Tissues were digested sequentially with 4 mg/mL protease (P5147, Sigma-Aldrich) for 90 min, followed by 0.25 mg/mL collagenase P (11213873,001, Roche, Mannheim, Germany) for 12 h. Isolated cells were cultured in a 5% CO₂ incubator at 37 °C. Chondrocytes were seeded into 75 cm^2^ culture flasks and maintained in DMEM/F-12 (Gibco Life Technologies) supplemented with 10% FBS (Gibco Life Technologies) and 1% penicillin–streptomycin (Gibco Life Technologies) at 37 °C with 5% CO₂. The chondrocytes were used in the experiments without dedifferentiation (Fig. S9a-b).

### RNA extraction, reverse transcription, and qRT-PCR

Total RNA was extracted from isolated chondrocytes utilizing the AG RNAex Pro reagent (cat. no. AG21101, Accurate Biotechnology, Hunan, China). Nuclear and cytoplasmic RNA were isolated following the manufacturer’s protocol using the Nuclear and Cytoplasmic Extraction Reagents Kit (cat. no. P0027, Beyotime Biotechnology, Beijing, China), as described previously. Reverse transcription was carried out using a PrimeScript RT reagent kit (cat. no. AG11706, Accurate Biotechnology, Hunan, China). RT-qPCR analysis was carried out using an ABI ViiA™ 7 Real-Time PCR System (Applied Biosystems, Foster City, CA, USA). The specific primers employed for these experiments are enumerated in Table S1. Gene expression levels were determined using the 2^−ΔΔCt^ method.

### Western blotting

Cell and tissue lysates were prepared using RIPA lysis buffer (cat. no.P0013E, Beyotime Biotechnology, China) containing protease inhibitors (cat. no.AB201111, 1:100, Abcam, Cambridge, UK) and phosphatase inhibitors (cat. no.AB201113, 1:100, Abcam, Cambridge, UK). Proteins were separated by SDS-PAGE and subsequently transferred to polyvinylidene difluoride membranes (cat. no.IPVH00010&ISEQ00010, Merck Millipore, Billerica, MA, USA). Electrophoresis was initiated at 80 V for 20 min, followed by 120 V for 1 h, and 250 mA transfer for 1.5 h. Membranes were blocked for 15 min with protein-free rapid blocking buffer (PS108P, Epizyme, Shanghai, China) before incubation with primary antibodies at 4 °C (Table S2). Following incubation, membranes were treated with the horseradish peroxidase-conjugated mouse secondary antibodies (cat. no.&7076, 1:3000, Cell Signaling Technology, Danvers, MA, USA) or rabbit secondary antibodies (cat. no.7074, 1:3000, Cell Signaling Technology, Danvers, MA, USA) at room temperature for 1 h and visualized using an ECL chemiluminescence kit (cat. no.WBKLS0500, Merck Millipore, Billerica, MA, USA). The intensity of bands was compared using ImageJ software (NIH, Bethesda, MD, USA).

### Cell transfection

Chondrocytes were seeded in six-well plates and transfected with circHIPK2 siRNAs (RiboBio, Guangzhou, China) or overexpression/knockdown plasmids constructed in pcDNA3.1 (Tsingke Biotechnology, China). These included circHIPK2, FTO, YTHDF2, and their corresponding wild-type, mutant (mut), or deletion (Del) variants. Transfection was performed using Lipofectamine 3000 (Invitrogen, USA). Cells were harvested 48 h post-transfection for qRT-PCR analysis and 72 h for Western blotting.

### Micro-CT analysis

Mouse knee joints were harvested and fixed in 4% paraformaldehyde. Samples were scanned on a Bruker SkyScan 2211 nano–computed tomography system (Bruker MicroCT, Kontich, Belgium) at an isotropic voxel size of 8.5 μm. Scans were acquired over 180° rotation at 90 kVp and 450 μA (8 W) with a 0.5-mm aluminum filter. Three-dimensional reconstructions were generated using InstaRecon (Bruker MicroCT, Kontich, Belgium). The femoral condyles, tibial plateau, and tibial shaft were defined as regions of interest (ROIs). Osteophyte volume and scoring were quantified using CTAn software (v1.20.8, Bruker MicroCT, Kontich, Belgium).

### RNA fluorescence in situ hybridization

The circHIPK2 fluorescent oligonucleotide probes were designed and synthesized by Servicebio (Wuhan, China). A RNA fluorescent in situ hybridization kit (cat. no. GF-003, Servicebio, China) was used to detect circHIPK2 expression in chondrocytes and knee joint sections. All images were acquired using a confocal microscope (LSM780; Carl Zeiss, Germany).

### Flow cytometry assay

Chondrocytes were seeded in six-well plates and cultured until they reached 80% confluence, then transfected with indicated plasmids. At 24 h post-transfection, chondrocytes were washed with cold PBS, dissociated into single-cell suspensions, and stained using a Cell Cycle Analysis Kit (cat. no. G1700, Servicebio, China). Flow cytometry results were analyzed with FlowJo v10 software.

### Histology, immunofluorescence, immunohistochemistry

Cartilage samples were fixed in 4% paraformaldehyde for 48 h, decalcified, embedded in paraffin, and cut into 5-µm sections. Sections were then stained with Safranin O/Fast Green (cat. no. G1053, Servicebio, China). For immunofluorescence, cells were fixed with 4% paraformaldehyde for 30 min, permeabilized with 0.5% Triton X-100 solution (cat. no. G1204, Servicebio, China) for 15 min, blocked with 5% Bovine Serum Albumin (BSA) for 30 min at room temperature, and then incubated with primary antibodies (Table S2) overnight at 4 °C. After washing three times with PBS, cells were incubated with specific fluorescent secondary antibody (cat. no. GB25401, GB25402, GB25403, GB25404, Servicebio, China) for 1 h at room temperature. Next, the nuclei were stained with DAPI (cat. no. G1012, Servicebio, China) for 8 min. All images were acquired randomly using a fluorescence inverted microscope (Leica, Germany). For immunohistochemistry, sections were incubated with 3% H_2_O_2_ to remove endogenous peroxidase. Antigen retrieval was performed with 0.1% trypsin without EDTA for 2 h at 37 °C. After blocking with 5% BSA for 1 h at room temperature, sections were incubated with primary antibodies (Table S2) overnight at 4 °C. Next day, the sections were incubated with peroxidase-conjugated secondary antibodies for 30 min (cat. no. G1213&G1214, Servicebio, China) and detected using DAB substrate (cat. no. G1202, Servicebio, China).

### Histological assessment

After safranin O/Fast green staining and HE staining. All sections were evaluated by Osteoarthritis Research Society International (OARSI) system and synovitis score to evaluate the OA degree by two independent individuals.

### γH2AX foci and cellular senescence β-Galactosidase staining

The γH2AX foci was analyzed to detect DNA damage. In brief, human chondrocytes were seeded on coverslips and cultured for 24 h at 37 °C in a humidified incubator with 5% CO₂. Cells were then fixed with 4% paraformaldehyde in PBS for 10 min, permeabilized with 0.25% Triton X-100 in PBS for 10 min, washed with PBS, and blocked for 1 h in 1% BSA (Sigma-Aldrich) prepared in PBS containing 0.1% Tween-20. γH2AX was incubated with a rabbit polyclonal antibody (Phospho-Histone H2A.X Ser139, Cell Signaling, 1:100 dilution, cat. no. 9718) overnight at 4 °C. After washing three times with PBS, cells were incubated with specific fluorescent secondary antibody (cat. no. GB25404, Servicebio, China) for 1 h at room temperature. The images were visualized by a confocal microscope (LSM 780, Zeiss, Oberkochen, Germany). For cellular senescence β-galactosidase staining, human chondrocytes were seeded in 6-well plates and stimulated with etoposide (20 μg/mL, 24 h) to induce senescence. Cells were washed with PBS and fixed with β-galactosidase fixative for 15 min at room temperature. After washing again with PBS, pre-warmed β-galactosidase staining solution containing X-Gal was added, and cells were incubated overnight at 37 °C. Following PBS rinses, senescent cells were observed under a brightfield microscope.

### RNA immunoprecipitation (RIP) assay

RIP assay was performed using Magna RIP™ RNA Binding Protein Immunoprecipitation kit (cat. no. 17–700, Millipore, USA). Co-precipitated RNAs were detected using RT-qPCR.

### RNA pull-down assay

Chondrocytes were harvested and lysed in lysis buffer, and total protein was collected. An aliquot containing 20 µg of total protein was reserved as input. The remaining lysate was split into two fractions and incubated overnight at 4 °C with streptavidin Dynabeads pre-bound to either a biotinylated circHIPK2-specific probe or a biotinylated control probe, using an RNA pull-down kit (cat. no. KT103, Gzscbio, Guangzhou, China). After pull-down, the captured proteins were eluted and subjected to mass spectrometry for identification. The probe sequences were as follows: circHIPK2 probes, CCATACCTGTAATATCTGGA-3'-biotin; Vector probe, CTGAATATCGACGGTTTCCA-3'-biotin.

### mRFP-GFP-LC3 adenovirus double label

Chondrocytes were seeded onto sterile glass coverslips in 24-well plates and cultured until approximately 70–80% confluence. mRFP-GFP-LC3 adenovirus (MOI = 50, Hanbio, Shanghai, China) was infected in 1 mL culture media for 4 h. Supplemented with 1 mL medium and cells were transfected for 8 h. Then, the medium was replaced with complete culture medium to incubate chondrocytes for 24 h. At room temperature, chondrocytes were fixed with 4% paraformaldehyde for 30 min. The nuclei were stained with DAPI for 10 min. Finally, the NIKON Eclipse Ti microscope (Tokyo, Japan) was used to obtain the images.

### Transmission electron microscopy (TEM)

Chondrocytes were fixed with 2.5% glutaraldehyde (pH 7.4, Sigma-Aldrich) for 2 h at room temperature. After low-speed centrifugation (1,200 rpm, 3 min), a compact cell pellet formed at the bottom of the tube. The pellet was fixed overnight in 2.5% glutaraldehyde, followed by post-fixation in 1% osmium tetroxide (Structure Probe, Inc., USA) for 2 h at 4 °C. Samples were then dehydrated, infiltrated, embedded, and sectioned, and ultrathin sections were imaged using a Tecnai G2 Spirit Twin transmission electron microscope (FEI, USA).

### LNP encapsulation of circHIPK2

CircHIPK2 was produced and purified by Geneseed Biotech (Guangdong, China) [47]. Next, circHIPK2 was encapsulated in LNPs following the previously described methods [48]. Briefly, the preparation steps included: (1) Encapsulation: circRNA was diluted in sodium acetate buffer (pH = 4.0) and was rapidly mixed with ethanolic lipid solution (a molar ratio of ALC-0315:DSPC:Cholesterol:ALC-0519 = 46.3:9.4:42.7:1.6) using a microfluidic device (Micro & Nano Biologics Co., Ltd.), maintaining a volume ratio of 3:1 for circRNA to lipids. (2) Purification: LNP–circRNA preparations were diluted in 1 × PBS (pH 7.2) and purified using 100-kDa ultrafiltration devices (Amicon Ultra centrifugal filter units; Millipore) at 4 °C. The Stunner system (Unchained Labs, USA) was used to measure the encapsulation efficiency and physicochemical properties of circHIPK2-loaded LNPs, and simultaneously quantify the particle size and zeta potential. The nanoparticles have a near-neutral zeta potential of –3.94 mV, a hydrodynamic diameter of 70.06 nm, and a polydispersity index (PDI) of 0.05, indicating a monodisperse and uniform preparation.

### Animal experiments

All animal experiments were conducted following the approval of the Institutional Animal Care and Use Committee, Sun Yat-Sen University. Experimental osteoarthritis was induced in 12-week-old male C57BL/6 mice. We randomly divided those mice into different groups (n = 5 per group). Before DMM surgery, animals were anesthetized using 1% sodium pentobarbital. The medial meniscotibial ligament was transected using microsurgical scissors, the joint cavity was irrigated with sterile saline, and the incision was closed with sutures. Sham-operated mice underwent skin incision and closure without transection of the medial meniscotibial ligament. After intra-articular injection of circHIPK2-LNP or PBS, the mice were sacrificed 4 weeks later. Blood samples were collected at 7 days post-injection for routine examination and biochemical analysis. The hearts, livers, spleens, lungs, and kidneys were photographed and subjected to hematoxylin–eosin (HE) staining. HanBio (Shanghai, China) created and packaged adeno-associated virus (AAV) vectors for circHIPK2 (mouse species, mmu_circ_0001468) and RAB22A (mouse species). One weeks after operation, a total of 10 μL (approximately 1.0 × 10^10^ vg) of vector AAV, RAB22A AAV, circHIPK2 AAV or LNP-circHIPK2 (0.375 mg/kg, followed by manufacture recommendation) was injected intra-articularly in the left knee once a week after surgery. Eight weeks post-DMM surgery, mice were euthanized, and knee joints were collected for micro-CT, HE staining, Safranin O-fast green staining, IHC staining, IF staining.

### Mouse gait analysis and pain assay

Gait changes were assessed using the Runway Scan 3.0 system (Clever Sys) as previously described [49]. Briefly, mice were placed individually on a 5-cm-wide runway and allowed to walk freely while gait was recorded with a high-speed camera. Swing times for each limb were captured, and the left hindlimb-to-right hindlimb swing-time ratio was calculated for analysis. OA-associated pain was evaluated using the von Frey and hot-plate assays as described previously [50].

### Statistical analysis

Statistical analyses were conducted in SPSS v23.0 (IBM, USA). Normality was evaluated using the Shapiro–Wilk test, and homogeneity of variance was examined with Levene’s test. For two-group comparisons, an unpaired two-tailed Student’s t-test was applied when data were normally distributed with equal variances; Welch’s t-test was used when variances were unequal; and the Mann–Whitney U test was used for non-normally distributed data. For comparisons among multiple groups, one-way ANOVA (normal distribution) or the Kruskal–Wallis test (non-normal distribution) was performed, followed by Bonferroni post hoc correction as appropriate. A two-sided P value < 0.05 was considered statistically significant.

## Supplementary Information

Supplementary Material 1.

## Data Availability

RNA-seq data have been deposited in the NCBI GEO database under accession codes 220,487 (https://www.ncbi.nlm.nih.gov/geo/query/acc.cgi?acc=GSE220487).
