# Supplementary material for: YTH N6-methyladenosine RNA binding protein 2 mediated m6A modification of circHIPK2 promotes cellular senescence and osteoarthritis progression by inhibiting autophagy
Source: Mol Biomed. 2026 Mar 27;7:39. doi: 10.1186/s43556-026-00441-4 (PMC13031695; doi:10.1186/s43556-026-00441-4)
Supplement: Supplementary file 1 — Supplementary Material 1. [file 43556_2026_441_MOESM1_ESM.docx]

**YTH N6-methyladenosine RNA binding protein 2 mediated m^6^A modification of circHIPK2 promotes cellular senescence and osteoarthritis progression by inhibiting autophagy**

Dianbo Long^1,^ ^3#^, Zhencan Lin^1, 3#^, Zhiwen Li^2, 3#^, Ming Li^1, 3#^, Xiaoyi Zhao^2, 3^, Zengfa Deng^1, 3^, Zongrui Jiang^1, 3^, Wei Li^1, 3^, Yanlin Zhong^1, 3^, Aishan He^1, 3^, Yiyang Xu^4*^, Guping Mao^1, 3*^, Yan Kang^1,3*^

**Affiliations:**

^1^ Department of Sports Medicine, the First Affiliated Hospital, Sun Yat-sen University, Guangzhou, Guangdong 510080, China.

^2^ Department of Joint Surgery, the First Affiliated Hospital, Sun Yat-sen University, Guangzhou, Guangdong 510080, China.

^3^ Guangdong Provincial Key Laboratory of Orthopedics and Traumatology, the First Affiliated Hospital, Sun Yat-sen University, Guangzhou, Guangdong 510080, China.

^4^ Department of Orthopaedics, Fujian Provincial Hospital; Shengli Clinical Medical College, Fuzhou University Affiliated Provincial Hospital, Fuzhou, Fujian 350001, China.

# These authors contributed equally to this work.

***Correspondence to:**

Yan Kang, Email: kangyan2@mail.sysu.edu.cn

Guping Mao, Email: maogp@mail2.sysu.edu.cn

Yiyang Xu, Email: fjxuyiyang@163.com

**Supplementary Figures**


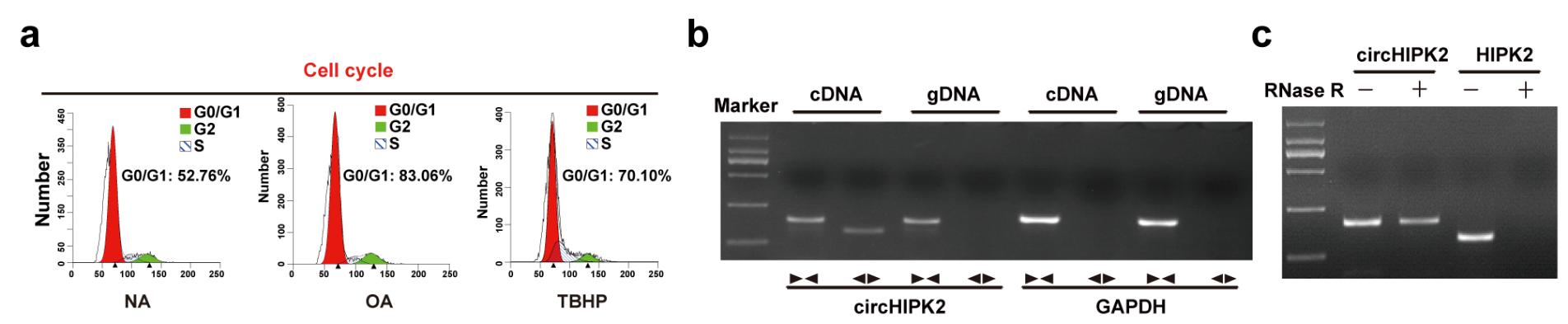


**Fig. S1 The expression levels of key chondrocyte markers in p0-p3 chondrocytes and circHIPK2 characteristics.** (**a**) Cell cycle analysis of chondrocytes by flow cytometry. (**b**) RT-qPCR analysis of circHIPK2 amplification using divergent primers and linear primers in chondrocytes. (**c**) CircHIPK2 and HIPK2 expression in chondrocytes treated with or without RNase R.


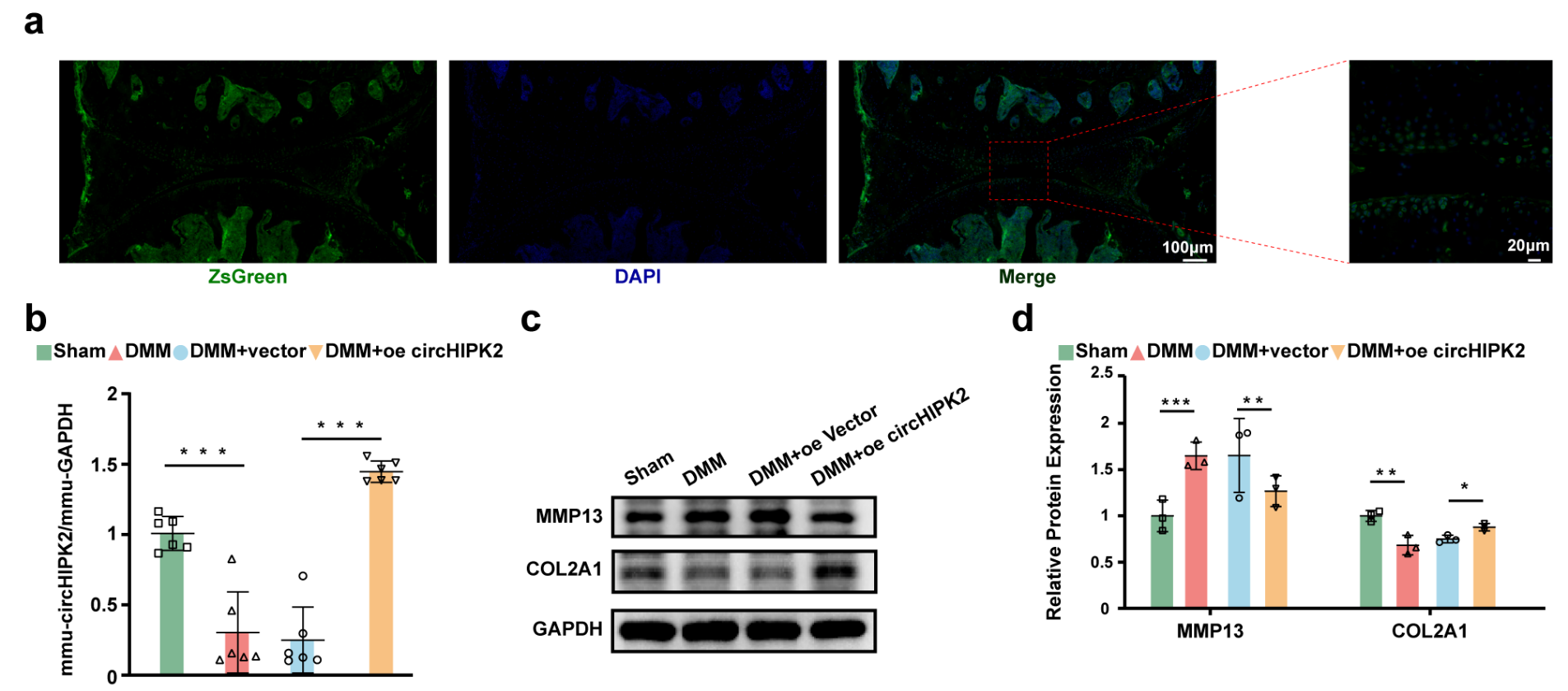


**Fig. S2 The biological effects of circHIPK2 in vivo.** (**a**) The fluorogram of adeno-associated virus (AAV) infecting the cartilage and synovium. (**b**) RT-qPCR analysis of circHIPK2 expression in mouse knee joint chondrocytes of each group. (**c**, **d**) Western blot analysis of COL2A1 and MMP13 protein levels in mouse knee joint chondrocytes of each group. **P* < 0.05, ***P* < 0.01, ****P* < 0.001.


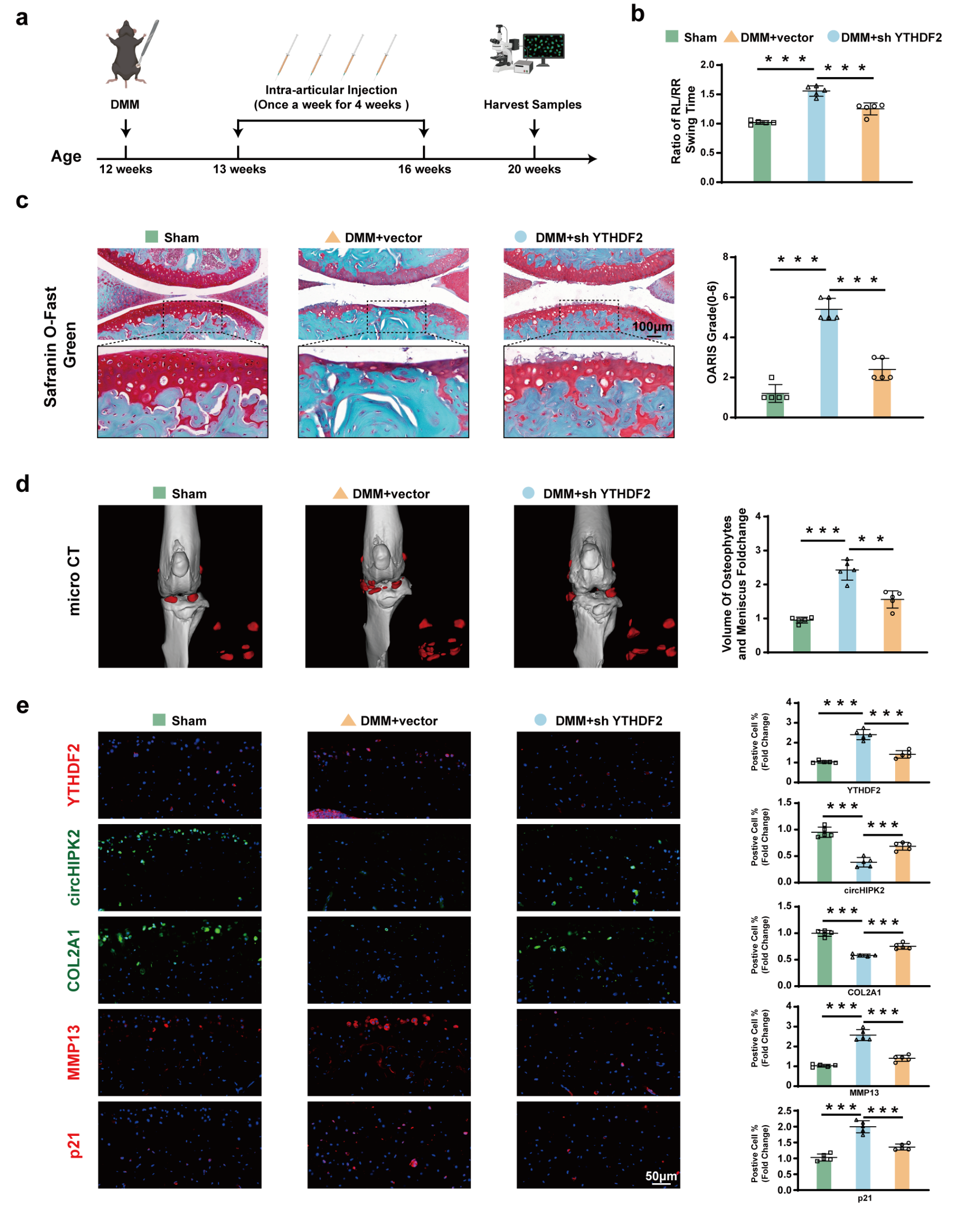


**Fig. S3 The biological role of YTHDF2 in vivo.** (**a**) Schematic illustration of the experimental design in the DMM mouse model (n=5 per group). (**b**) Ratio of rear left (RL) to rear right (RR) limb swing time of mice in each group according to gait analysis. (**c**) S-O-G staining was performed to assess the cartilage degeneration. (**d**) Micro-CT images of knee joint calcified meniscus and osteophytes (Red) and quantification of the volume in each group. (**e**) IF staining and FISH of YTHDF2, circHIPK2, COL2A1, MMP13 and p21 in the knee joints of mice in the three groups. ^**^*P* < 0.01, ^***^*P* < 0.001.


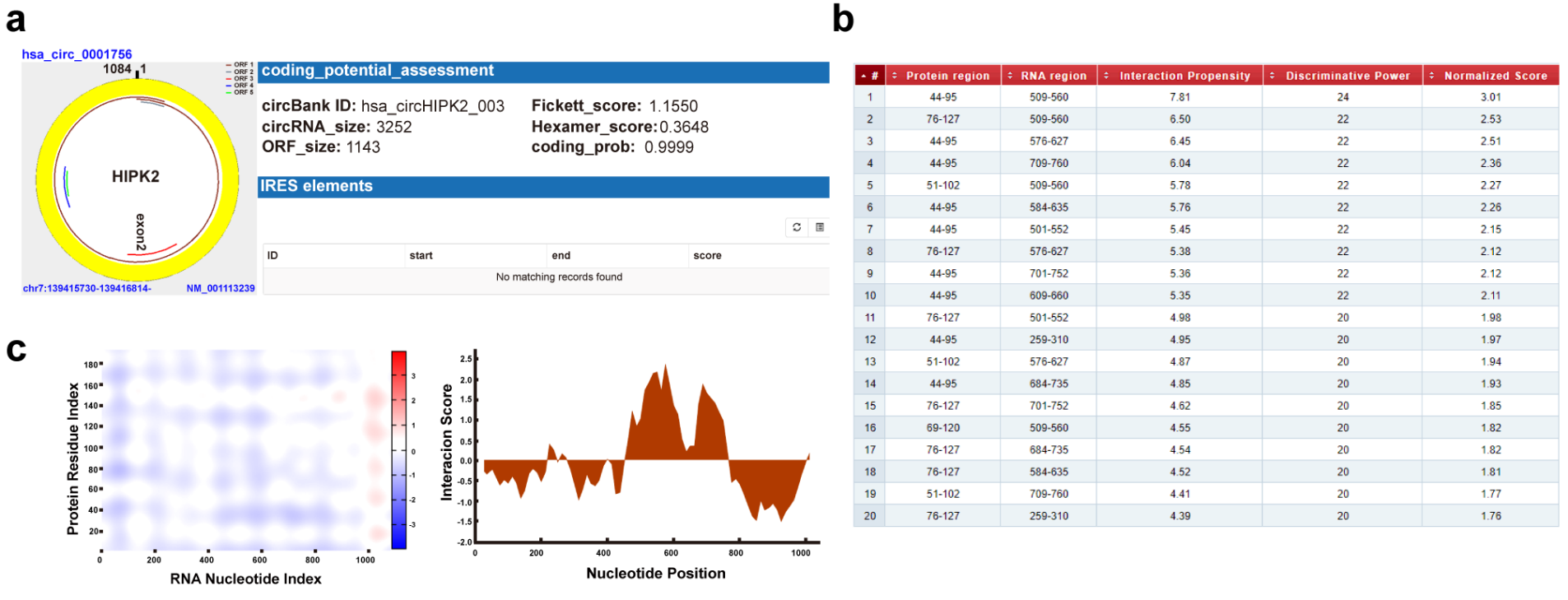


**Fig. S4 The prediction of translation potential of circHIPK2.** (**a**) CircPrimer2.0 software and CircBank database indicated the possibility of encoding proteins of CircHIPK2. (**b, c**) The interaction between CircHIPK2 and RAB22A was predicted using the CatRAPID.


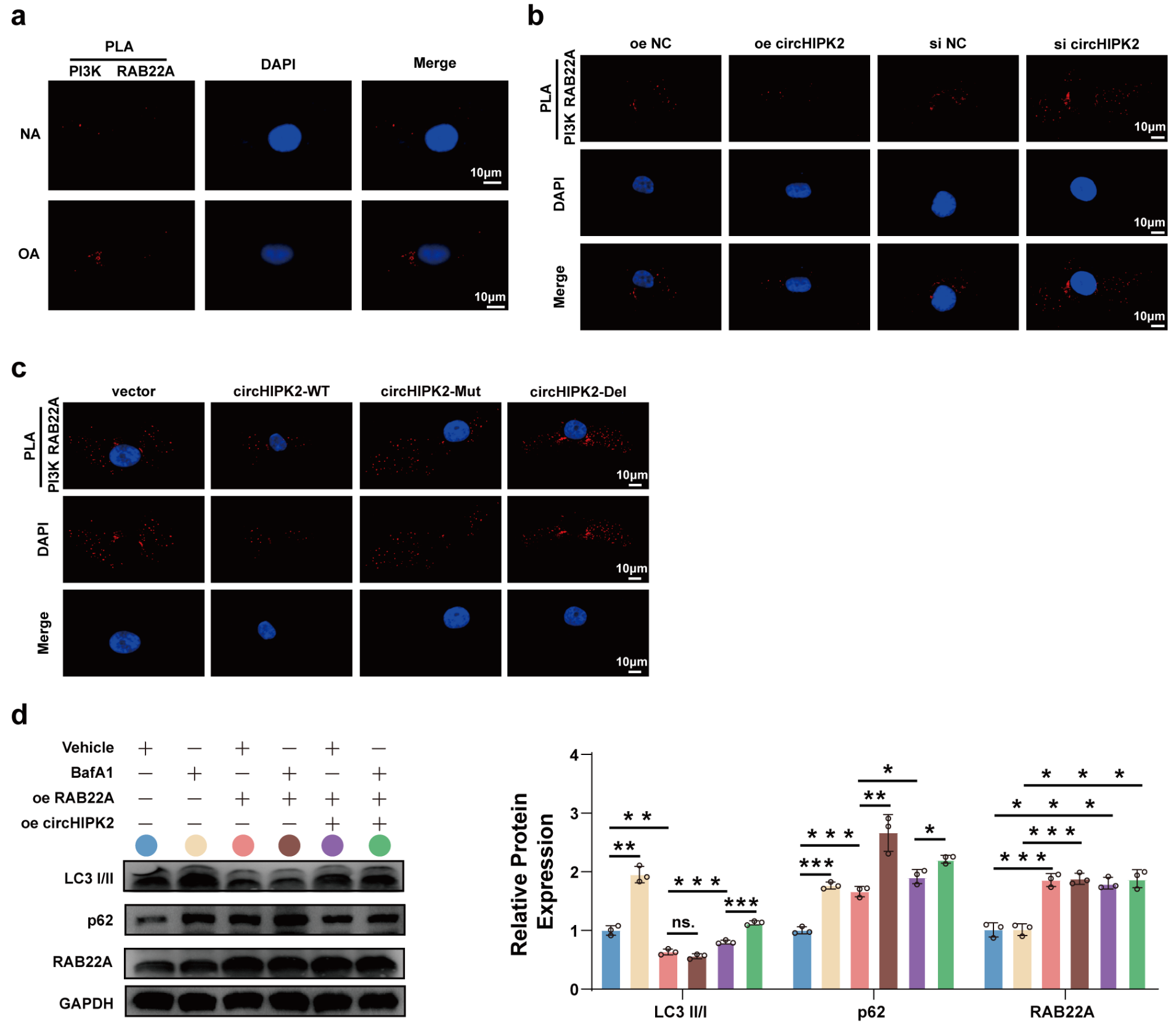


**Fig. S5 CircHIPK2 inhibits autophagy via regulating the interaction between RAB22A and PI3K.** (**a**) Proximity ligation assays (PLA) were performed to evaluate the interaction between RAB22A and PI3K in normal and OA chondrocytes. (**b**) PLA analysis of the proximity between RAB22A and PI3K in chondrocytes transfected with oe circHIPK2, si circHIPK2, or the negative controls plasmids. (**c**) PLA analysis of the proximity between RAB22A and PI3K in chondrocytes transfected with circHIPK2–WT, circHIPK2–Mut, circHIPK2–Del or the negative controls plasmids. (**d**) Chondrocytes were treated with BafA1 (100 nM) and transfected with RAB22A overexpression plasmids, circHIPK2 overexpression plasmids, or the corresponding negative controls. ^*^*P* < 0.05, ^**^*P* < 0.01, ^***^*P* < 0.001.


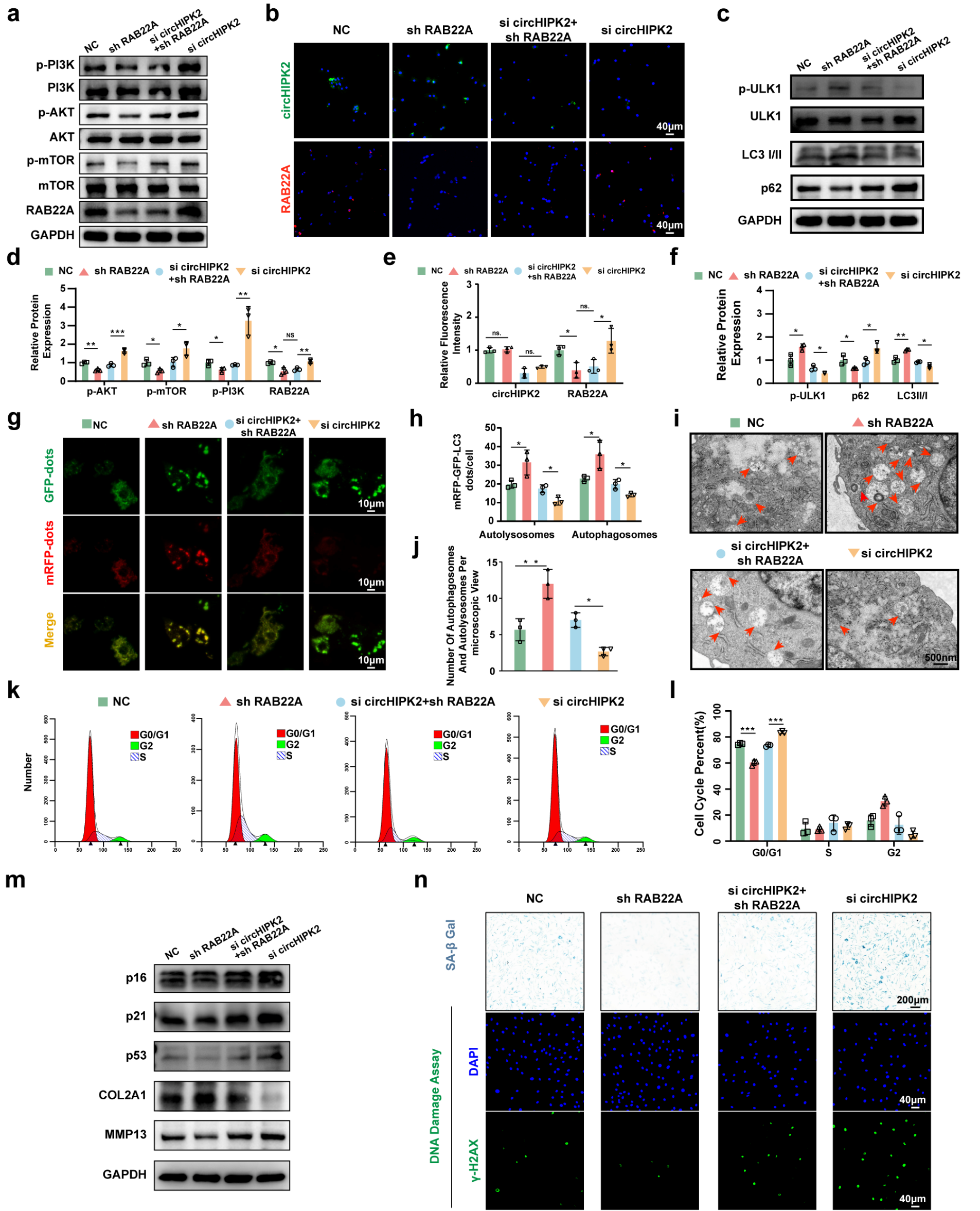


**Fig. S6 CircHIPK2 inhibits chondrocyte senescence by promoting autophagy through RAB22A.** Chondrocytes were transfected with si circHIPK2, sh RAB22A or negative control plasmids. (**a**) Western blot analysis of p-PI3K, p-AKT, p-mTOR, PI3K, AKT, and mTOR proteins. (**b**) FISH and immunofluorescence analysis of circHIPK2 and RAB22A levels. (**c**) Western blot analysis of autophagy-associated proteins p-ULK1, ULK1, P62, LC3 I/II, BCL2, and BAX. (**d-f**) Quantification of western blotting, FISH and immunofluorescence analysis. (**g-h**) mRFP-GFP-LC3 double-labeled autophagy adenovirus was employed to assess changes in autophagic flux in chondrocytes. (**i-j**) Representative TEM images of autophagosomes and autolysosomes in chondrocytes. Green arrows: autophagosomes, red arrows: autolysosomes. (**k-l**) Cell cycle analysis of chondrocytes by flow cytometry. (**m**) Western blot analysis of chondrocytes extracellular matrix-associated proteins and senescence-associated proteins. (**n**) γH2AX and SA-β-gal staining of chondrocytes. GAPDH served as control. ^*^*P* < 0.05, ^**^*P* < 0.01, ^***^*P* < 0.001, ns: no significance.


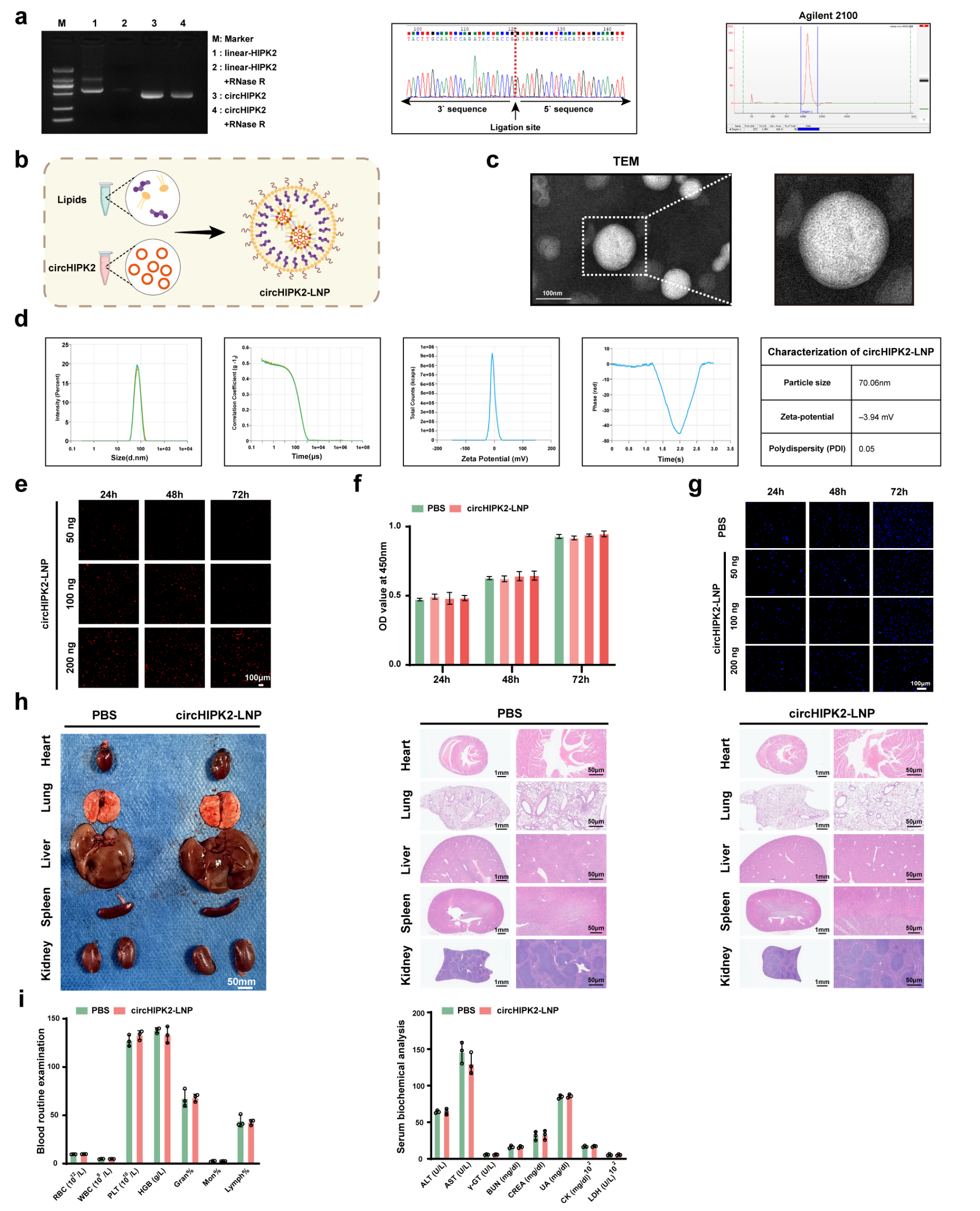


**Fig. S7 Synthesis and characterization of circHIPK2-LNP.** (**a**) The generation and identification of circHIPK2 in vitro. (**b**) Schematic of circHIPK2-LNP. (**c**) TEM image of circHIPK2-LNP. (**d**) The diameter and zeta potential of circHIPK2-LNP. (**e**) Representative fluorescent images of chondrocytes transfected with different doses of cy5-circHIPK2-LNP at different time points. (**f-g**) CCK-8 assay and TUNEL assay were used to assess chondrocyte death. (**h**) The general appearance and H&E-stained tissue sections of major organs at 4 weeks after intra-articular injection of circHIPK2-LNP. (**i**) Blood routine examination and serum biochemical analysis at 7 days after intra-articular injection of circHIPK2-LNP.


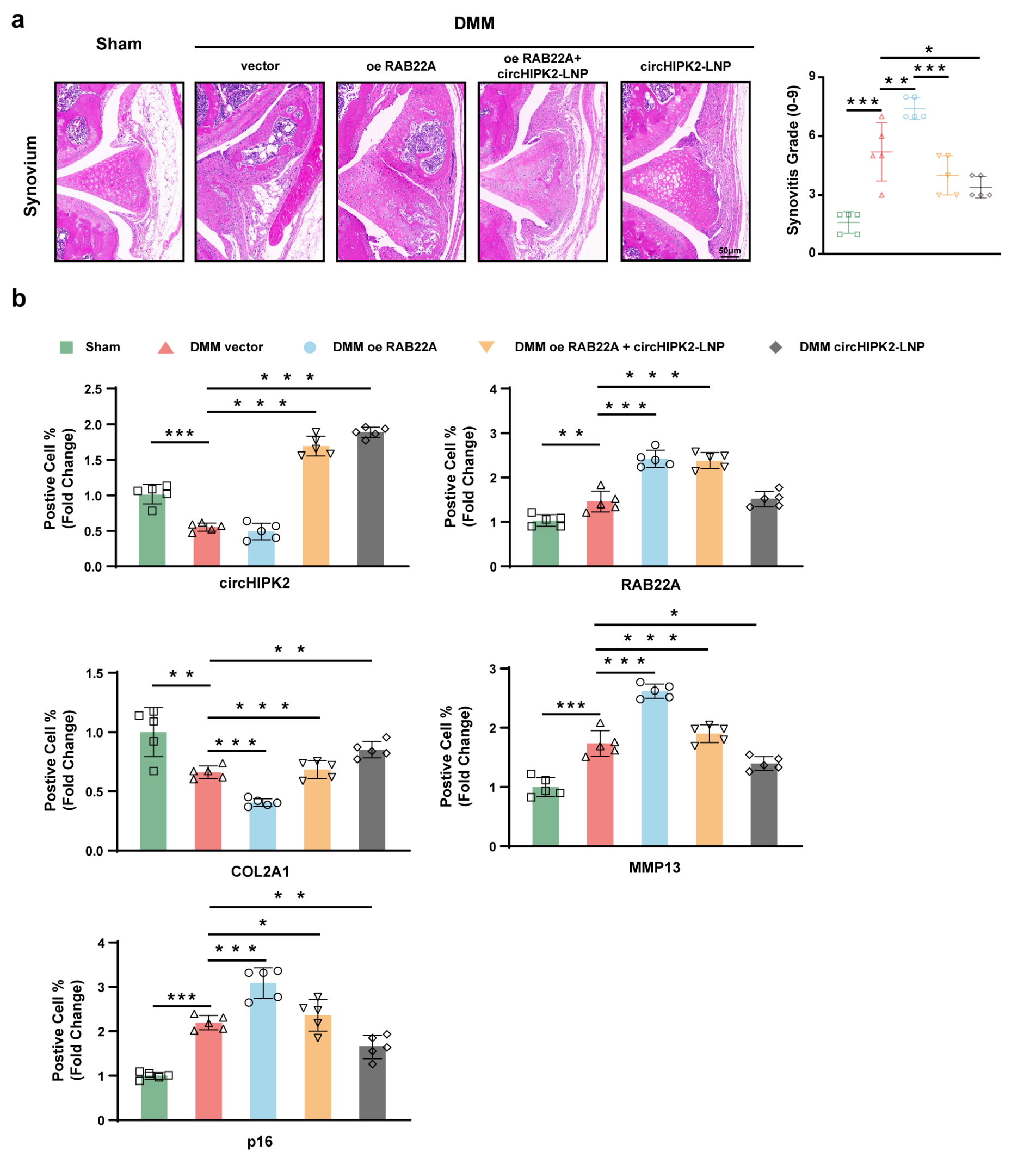


**Fig. S8 Therapeutic effect of circHIPK2-LNP in OA mice.** (**a**) HE staining was performed to assess the synovial hyperplasia in each group. (**b**) Immunohistochemistry staining, immunofluorescence staining and FISH of circHIPK2, RAB22A, COL2A1, MMP13, and P16 in the knee joints of mice in each group. ^*^*P* < 0.05, ^**^*P* < 0.01, ^***^*P* < 0.001.


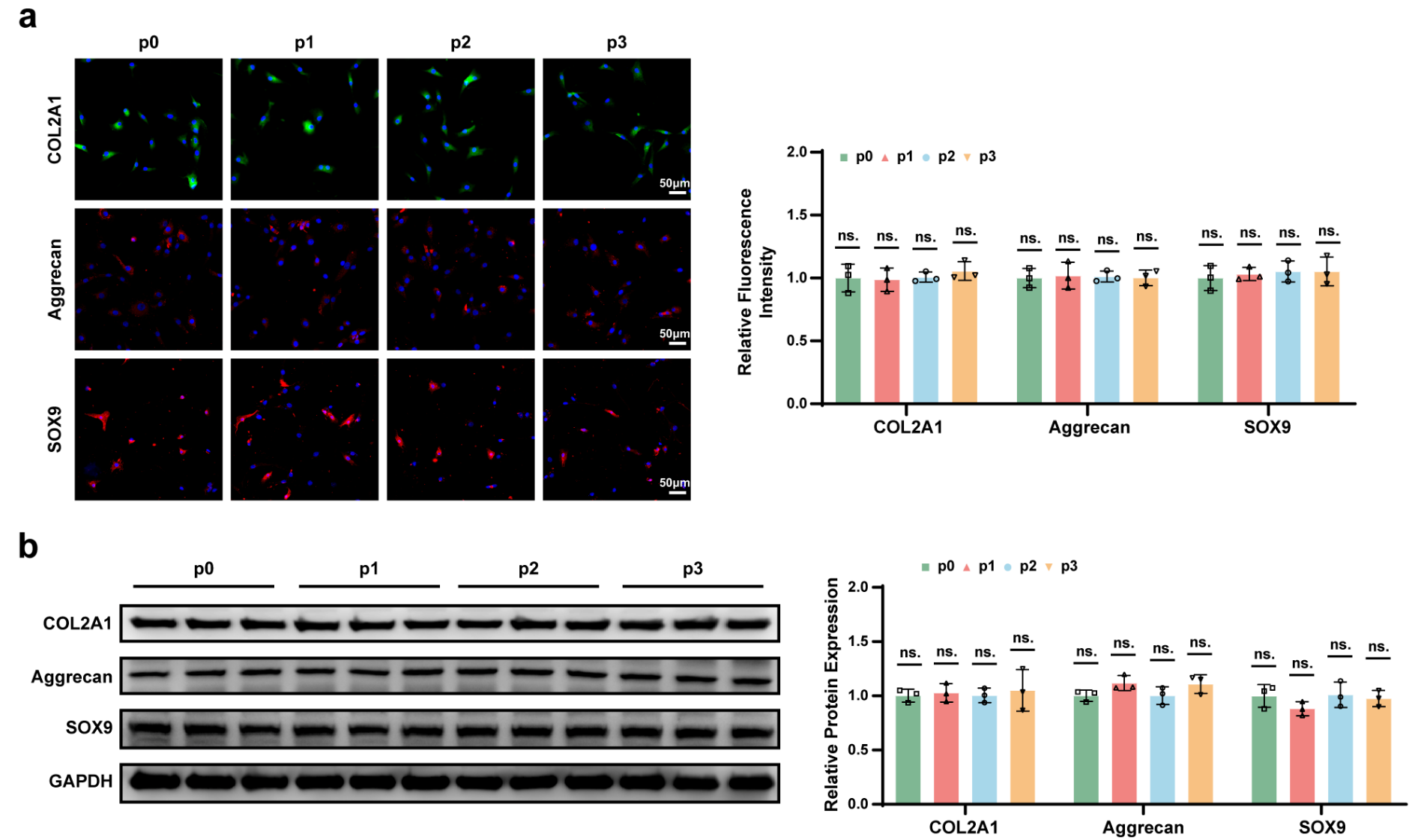


**Fig. S9 The expression levels of key chondrocyte markers in p0-p3 chondrocytes. (a-b)** Immunofluorescence and WB analysis were performed to evaluate the expression levels of classic chondrocyte markers (COL2A1, SOX9, and Aggrecan) in chondrocytes at passage 0, 1, 2, and 3. ns: no significance.

**Supplemental information**

**CircHIPK2 purified sequence**

GTATGGCCTCACATGTGCAAGTTTTCTCCCCTCACACCCTTCAATCAAGTGCCTTCTGTAGTGTGAAGAAACTGAAAATAGAGCCGAGTTCCAACTGGGACATGACTGGGTACGGCTCCCACAGCAAAGTGTATAGCCAGAGCAAGAACATCCCCCTGTCGCAGCCAGCCACCACAACCGTCAGCACCTCCTTGCCGGTCCCAAACCCAAGCCTACCTTACGAGCAGACCATCGTCTTCCCAGGAAGCACCGGGCACATCGTGGTCACCTCAGCAAGCAGCACTTCTGTCACCGGGCAAGTCCTCGGCGGACCACACAACCTAATGCGTCGAAGCACTGTGAGCCTCCTTGATACCTACCAAAAATGTGGACTCAAGCGTAAGAGCGAGGAGATCGAGAACACAAGCAGCGTGCAGATCATCGAGGAGCATCCACCCATGATTCAGAATAATGCAAGCGGGGCCACTGTCGCCACTGCCACCACGTCTACTGCCACCTCCAAAAACAGCGGCTCCAACAGCGAGGGCGACTATCAGCTGGTGCAGCATGAGGTGCTGTGCTCCATGACCAACACCTACGAGGTCTTAGAGTTCTTGGGCCGAGGGACGTTTGGGCAAGTGGTCAAGTGCTGGAAACGGGGCACCAATGAGATCGTAGCCATCAAGATCCTGAAGAACCACCCATCCTATGCCCGACAAGGTCAGATTGAAGTGAGCATCCTGGCCCGGTTGAGCACGGAGAGTGCCGATGACTATAACTTCGTCCGGGCCTACGAATGCTTCCAGCACAAGAACCACACGTGCTTGGTCTTCGAGATGTTGGAGCAGAACCTCTATGACTTTCTGAAGCAAAACAAGTTTAGCCCCTTGCCCCTCAAATACATTCGCCCAGTTCTCCAGCAGGTAGCCACAGCCCTGATGAAACTCAAAAGCCTAGGTCTTATCCACGCTGACCTCAAACCAGAAAACATCATGCTGGTGGATCCATCTAGACAACCATACAGAGTCAAGGTCATCGACTTTGGTTCAGCCAGCCACGTCTCCAAGGCTGTGTGCTCCACCTACTTGCAGTCCAGATATTACAG

**Supplemental Table**

| **Table S1. Primers and sequences used in this study.** | |
| --- | --- |
| **Gene** | **Primer sequence (5’-3’)** |
| hsa-GAPDH-F | GGAGCGAGATCCCTCCAAAAT |
| hsa-GAPDH-R | GGCTGTTGTCATACTTCTCATGG |
| hsa-circ-HIPK2-F | AGGTCTTATCCACGCTGACC |
| hsa-circ-HIPK2-R | GAAGGGTGTGAGGGGAGAAA |
| hsa-circ-GOSR2-F | GGAAGGTCTGTGATTTGGCC |
| hsa-circ-GOSR2-R | TGTTCTTTGTCGGAGGGTGA |
| hsa-circ-REPS1-F | TATTTGGTTCTTCTGCTGGTGA |
| hsa-circ-REPS1-R | TTCGTACTGTTGTCTGGTCCT |
| hsa-circ-MTUS1-F | TATCCAAGCCTGACTCCTGC |
| hsa-circ-MTUS1-R | TGATTTGTTGTTCCCAGAACTG |
| hsa-circ-NUP54-F | TGCCAGATGATCAGTGGGTT |
| hsa-circ-NUP54-R | CCTCCAAATCCCAGTCCAGT |
| hsa-circ-SLC8A1-F | CATTGGCATCATGGAGGTGA |
| hsa-circ-SLC8A1-R | ACTTCCAACTGTCACAACACA |
| hsa-circ-ADAMTS6-F | GAACTATATTGGTCAGGTTGCCA |
| hsa-circ-ADAMTS6-R | GCATTATCGTGGTGGGCAAT |
| hsa-circ-TNFRSF21-F | ACTACTGCAATGGCCATGCTTGGA |
| hsa-circ-TNFRSF21-R | GCTGGACACTTGTCACAGGTTAG |
| hsa-circ-FN1-F | CCACCACACCCAATTCCTTG |
| hsa-circ-FN1-R | GGAGGAGGAACAGCTGTCTT |
| hsa-circ-APBB2-F | CCGGATGAAACAGCTGACTC |
| hsa-circ-APBB2-R | CATTTGGGAGGTGTGCTGTT |
| hsa-HIPK2-F | CCACCTACTTGCAGTCCAGA |
| hsa-HIPK2-R | AGCTCCTGGATATAACGGCC |
| hsa-METTL3-F | TTGTCTCCAACCTTCCGTAGT |
| hsa-METTL3-R | CCAGATCAGAGAGGTGGTGTAG |
| hsa-METTL14-F | AGTGCCGACAGCATTGGTG |
| hsa-METTL14-R | GGAGCAGAGGTATCATAGGAAGC |
| hsa-WTAP-F | CTTCCCAAGAAGGTTCGATTGA |
| hsa-WTAP-R | TCAGACTCTCTTAGGCCAGTTAC |
| hsa-FTO-F | ACTTGGCTCCCTTATCTGACC |
| hsa-FTO-R | TGTGCAGTGTGAGAAAGGCTT |
| hsa-ALKBH5-F | CGGCGAAGGCTACACTTACG |
| hsa-ALKBH5-R | CCACCAGCTTTTGGATCACCA |
| hsa-YTHDC1-F | AACTGGTTTCTAAGCCACTGAGC |
| hsa-YTHDC1-R | GGAGGCACTACTTGATAGACGA |
| hsa-YTHDF3-F | TCAGAGTAACAGCTATCCACCA |
| hsa-YTHDF3-R | GGTTGTCAGATATGGCATAGGCT |
| hsa-eLF3-F | GGCCGATGACTTGGTACTGAC |
| hsa-eLF3-R | GCTTGCGTCGTACTTGTTCTTC |
| hsa-YTHDF1-F | ATACCTCACCACCTACGGACA |
| hsa-YTHDF1-R | GTGCTGATAGATGTTGTTCCCC |
| hsa-YTHDF2-F | AGCCCCACTTCCTACCAGATG |
| hsa-YTHDF2-R | TGAGAACTGTTATTTCCCCATGC |
| hsa-COL2A1-F | CCAGATGACCTTCCTACGCC |
| hsa-COL2A1-R | TTCAGGGCAGTGTACGTGAAC |
| hsa-MMP13-F | CCAGACTTCACGATGGCATTG |
| hsa-MMP13-R | GGCATCTCCTCCATAATTTGGC |
| hsa-Aggrecan-F | GATGTTCCCTGCAATTACCACCTC |
| hsa-Aggrecan-R | TGATCTCATACCGGTCCTTCTTCTG |
| hsa-RUNX2-F | CACTGGCGCTGCAACAAGA |
| hsa-RUNX2-R | CATTCCGGAGCTCAGCAGAATAA |
| hsa-ADAMTS4-F | GAGGAGGAGATCGTGTTTCCA |
| hsa-ADAMTS4-R | CCAGCTCTAGTAGCAGCGTC |
| hsa-SOX9-F | AGCGAACGCACATCAAGAC |
| hsa-SOX9-R | CTGTAGGCGATCTGTTGGGG |
| mmu-GAPDH-F | TGTGTCCGTCGTGGATCTGA |
| mmu-GAPDH-R | TTGCTGTTGAAGTCGCAGGAG |
| mmu-circ-HIPK2-F | GCAATCCAGATACTACCGGT |
| mmu-circ-HIPK2-R | CAGTTGGAACTTGGCTCTAC |

| **Table S2. Primary antibodies used in this study.** | |
| --- | --- |
| **Primary antibodies** | **Article number and manufacturer** |
| m^6^A | ab286164, Abcam, UK |
| IgG | Ab172730, Abcam, UK |
| YTHDF2 | 24744-1-AP, Proteintech, USA |
| FTO | 27226-1-AP, Proteintech, USA |
| MMP13 | GB11247, Servicebio, China |
|  | GB11247-1, Servicebio, China |
| COL2A1 | ab188570, Abcam, UK |
|  | GB11021, Servicebio, China |
| RUNX2 | GB13264, Servicebio, China |
| Aggrecan | 13880-1-AP, Proteintech, USA |
| ADAMTS4 | 11865-1-AP, Proteintech, USA |
| SOX9 | GB115434, Servicebio, China |
| GAPDH | HRP-60004, Proteintech, USA |
| p-PI3K | 4228, Cell Signaling Technology, USA |
| PI3K | 4257, Cell Signaling Technology, USA |
| p-AKT | 4060, Cell Signaling Technology, USA |
| AKT | 4685, Cell Signaling Technology, USA |
| p-mTOR | 5536, Cell Signaling Technology, USA |
| mTOR | 2983, Cell Signaling Technology, USA |
| p-ULK1 | 80218-1-RR, Proteintech, USA |
| ULK1 | 20986-1-AP, Proteintech, USA |
| SQSTM1-p62 | GB11531, Servicebio, China |
| LC3-I/II | 43566, Cell Signaling Technology, USA |
| BCL2 | GB113375, Servicebio, China |
| BAX | GB114122, Servicebio, China |
| P16 | GB111605, Servicebio, China |
| P21 | GB115313, Servicebio, China |
| P53 | GB11626, Servicebio, China |
| RAB22A | 12125-1-AP, Proteintech, USA |

| **Table S3. Baseline characteristics patients with osteoarthritis (n = 30).** | |
| --- | --- |
| **Variables** | **Counts (%)** |
| **Age** |  |
| <65 | 10 (33.3) |
| ≥65 | 20 (66.7) |
| **Sex** |  |
| Male | 5 (16.7) |
| Female | 25 (83.3) |
| **Body Mass Index** |  |
| <18.5 | 0 (0.0) |
| ≥18.5, <24 | 6 (20.0) |
| ≥24, <28 | 16 (53.3) |
| ≥28 | 8 (26.7) |
| **Affected side** |  |
| Left | 12 (40) |
| Right | 18 (60) |
| **Disease duration (years)** |  |
| <10 | 14 (46.7) |
| ≥10 | 16 (53.3) |
| **Kellgren–Lawrence grade** |  |
| III | 12 (40) |
| IV | 18 (60) |

| **Table S4. The expression of m6A-related genes.** | | | | | | | | | | | | | | |
| --- | --- | --- | --- | --- | --- | --- | --- | --- | --- | --- | --- | --- | --- | --- |
| **Gene name** | **Relative expression** | | | | | | | | | | | | **p-values** | |
|  | **NA** | | | | | | **OA** | | | | | |  |  |
| FTO | 1.221754 | 1.076852 | 0.934977 | 0.933662 | 0.933373 | 0.932855 | 0.700855 | 0.684606 | 0.634543 | 0.54177 | 0.393594 | 0.354623 | 0.000170874 |  |
| METTL14 | 1.070692 | 0.959908 | 0.877808 | 1.540222 | 1.262462 | 0.570039 | 1.303845 | 1.300761 | 1.084208 | 1.183155 | 1.043365 | 1.214737 | 0.345426704 |  |
| METTL3 | 1.586391 | 0.860858 | 0.986637 | 1.117423 | 0.805289 | 0.824767 | 0.795875 | 0.749045 | 0.584939 | 0.925043 | 1.511937 | 0.595432 | 0.38126542 |  |
| WTAP | 1.144065 | 1.617754 | 0.992631 | 0.939076 | 0.814478 | 0.711654 | 2.152 | 1.911892 | 1.065195 | 1.60973 | 1.591137 | 1.210496 | 0.026297273 |  |
| ALKBH5 | 1.143282 | 1.095123 | 1.064848 | 0.988255 | 0.978903 | 0.775332 | 0.888297 | 0.858228 | 0.84642 | 0.834364 | 0.918188 | 0.976326 | 0.061705207 |  |
| ELF3 | 1.765087 | 1.666294 | 1.44591 | 0.802978 | 0.592648 | 0.494129 | 1.760488 | 1.642866 | 0.807812 | 0.611117 | 0.51621 | 0.458262 | 0.635906633 |  |
| YTHDF1 | 1.135859 | 0.969122 | 0.902542 | 0.934952 | 1.014101 | 1.061597 | 0.997196 | 1.1074 | 0.994842 | 1.1445 | 1.09844 | 1.113274 | 0.126634832 |  |
| YTHDF2 | 1.236217 | 1.128571 | 1.09096 | 1.071685 | 0.942464 | 0.650483 | 1.924694 | 1.825105 | 1.662169 | 1.568154 | 1.472741 | 1.349669 | 0.000494104 |  |
| YTHDF3 | 1.199888 | 1.101523 | 0.873127 | 0.677978 | 1.145148 | 1.116121 | 1.257896 | 1.167061 | 0.448362 | 0.26512 | 0.79898 | 0.7392 | 0.209720274 |  |
| YTHDC1 | 1.128196 | 1.17766 | 1.057872 | 0.993105 | 0.870427 | 0.823066 | 0.989315 | 0.866256 | 1.222252 | 1.071456 | 1.154615 | 1.462422 | 0.268669488 |  |
| YTHDC3 | 0.99937 | 1.043729 | 0.958331 | 0.936039 | 1.055597 | 1.012461 | 1.079887 | 1.190965 | 0.934164 | 1.040303 | 1.208647 | 1.390464 | 0.065344867 |  |
| COL2A1 | 1.270822 | 1.136847 | 1.070129 | 1.115268 | 0.789588 | 0.734509 | 1.056964 | 0.83545 | 0.723709 | 0.679719 | 0.54927 | 0.508291 | 0.033125252 |  |
| MMP13 | 1.081987 | 1.072781 | 1.06746 | 0.972666 | 0.949854 | 0.873564 | 2.1161 | 1.956777 | 1.81015 | 1.681053 | 1.651784 | 1.568716 | 5.59E-06 |  |
